# Synthesis and anion recognition properties of shape-persistent binaphthyl-containing chiral macrocyclic amides

**DOI:** 10.3762/bjoc.8.109

**Published:** 2012-06-28

**Authors:** Marco Caricato, Nerea Jordana Leza, Claudia Gargiulli, Giuseppe Gattuso, Daniele Dondi, Dario Pasini

**Affiliations:** 1 Department of Chemistry, University of Pavia, Viale Taramelli 10, 27100 Pavia, Italy; 2 Department of Organic and Biological Chemistry, University of Messina, Viale F. Stagno d'Alcontres 31, 98166 Messina, Italy; 3 INSTM Research Unit, Department of Chemistry, University of Pavia, 27100 Pavia, Italy

**Keywords:** amides, anion recognition, chirality, macrocycles, molecular switches, supramolecular chemistry

## Abstract

We report on the synthesis and characterization of novel shape-persistent, optically active arylamide macrocycles, which can be obtained using a one-pot methodology. Resolved, axially chiral binol scaffolds, which incorporate either methoxy or acetoxy functionalities in the 2,2' positions and carboxylic functionalities in the external 3,3' positions, were used as the source of chirality. Two of these binaphthyls are joined through amidation reactions using rigid diaryl amines of differing shapes, to give homochiral tetraamidic macrocycles. The recognition properties of these supramolecular receptors have been analyzed, and the results indicate a modulation of binding affinities towards dicarboxylate anions, with a drastic change of binding mode depending on the steric and electronic features of the functional groups in the 2,2' positions.

## Introduction

Macrocyclic molecules possessing a high degree of shape persistency act as molecular cages, and the scientific interest for such compounds is certainly increasing [1–4]. Support for this statement arises from consideration in two main areas of interest: (a) the recognition properties towards suitable guests are usually enhanced by limiting the number of conformations accessible to the covalent cyclic structure (resulting in preorganization [5]); (b) shape persistency is a requirement for the formation of organic nanotubes by means of supramolecular organization of macrocycles in the third dimension [6–12].

Amide functionalities are hydrogen-bonding tools of widespread use for the conformational stabilization of nanostructures. Noticeable examples can be found in the field of foldamers [13–14] or in the design of assembled architectures functioning as artificial ion-channel mimics [15]. Amide functionalities are also widely used for their hydrogen bonding capability in the context of anion complexation. Several macrocyclic systems capable of effective anion recognition and discrimination have been previously reported [16–18]. Binol (1,1'-binaphthyl-2,2'-diol) based synthons are popular in the recent literature; given their robustness, they are frequently used to impart or transfer chiral information, not only in the field of asymmetric synthesis and catalysis, but also in materials science [19–24].

**Figure 1:**
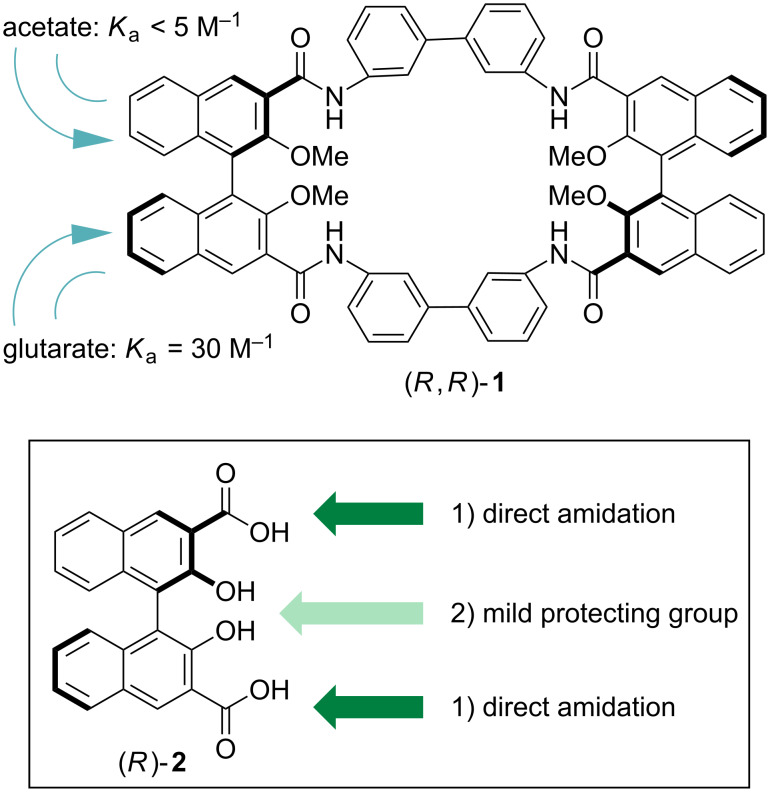
Structure of the macrocycle (*R*,*R*)-**1** (top), and synthetic strategies for the production of novel amide-containing, axially chiral macrocycles (bottom).

During the course of our ongoing efforts dealing with the use of binol-based synthons for the production of functional, oriented nanomaterials and chiroptical sensors [25–30], we have reported on the design, synthesis and characterization of a rigid, optically active tetraamidic macrocycle with recognition capabilities towards anions (Figure 1) [31].

In fact, macrocycle (*R*,*R*)-**1** could be obtained efficiently (62% in the macrocyclization step) through a sequential, convergent methodology. It is a 32-membered macrocycle whose cyclic backbone is composed exclusively of sp^2^-hybridized carbon and nitrogen atoms. An additional internal rigidification of the macrocyclic cavity is given by the presence of stable, six-membered intramolecular hydrogen bonds between the protected (in the form of methyl ether) phenol moieties in the 2,2' positions and the NH protons of the amide functionalities in the neighboring 3,3' positions of the binaphthyl units. Macrocycle (*R*,*R*)-**1** showed modest binding affinities towards carboxylate anions, yet detectable binding of proper difunctional carboxylates.

We deemed it to be very interesting to increase the availability of hydrogen-bond donors within the macrocycle cavity, and to unlock the hydrogen-bonding capability of the amide NHs to their full potential for anion recognition. The former could in principle be achieved by unmasking the phenolic oxygen atoms in the 2,2' positions of the binaphthyl skeletons. As for the latter, the introduction of another protecting group, sterically and electronically modulating the hydrogen-bond accepting capability of the phenolic oxygen, was needed.

In this paper, we present the exploitation of these strategies, resulting in the synthesis and characterization of three novel binaphthyl-based macrocycles, and the evaluation of their potential as supramolecular receptors for aliphatic bidentate carboxylate anions.

## Results and Discussion

### Design, synthesis and spectroscopic characterization

Reactions to deprotect the phenolic oxygens, performed directly on (*R*,*R*)-**1** as a substrate and attempted under various reaction conditions, proved completely unsuccessful with degradation of the macrocyclic structure occurring in all cases. On the basis of the introductory considerations mentioned earlier, we set out to exploit two orthogonal synthetic strategies (Figure 1, bottom): (1) A direct amidation of the carboxylic acids in the 3,3' positions, in the presence of the free phenolic oxygens in the 2,2' positions. Literature precedents for such amidation using aromatic amines in the presence of vicinal phenol moieties (which compete since they are comparable in nucleophilicity with aromatic amines) are rare [32–33]. As already reported [31], test reactions on model compounds gave disappointing results. The use of benzylic amines, more nucleophilic than arylamines, and therefore competing less with the phenolic moieties in the 2,2' positions, was envisaged as a potential solution and was therefore actively pursued. (2) The use of milder (with respect to methyl ether) protecting groups for the phenolic functionalities in the 2,2' positions; we focused on the use of compound **3**, bearing acetyl protecting groups, since its synthesis has been reported, and the deprotection of these groups usually occurs under mild basic conditions [34]. Aromatic amines, as in (*R*,*R*)-**1**, could in principle be used.

**Scheme 1:**
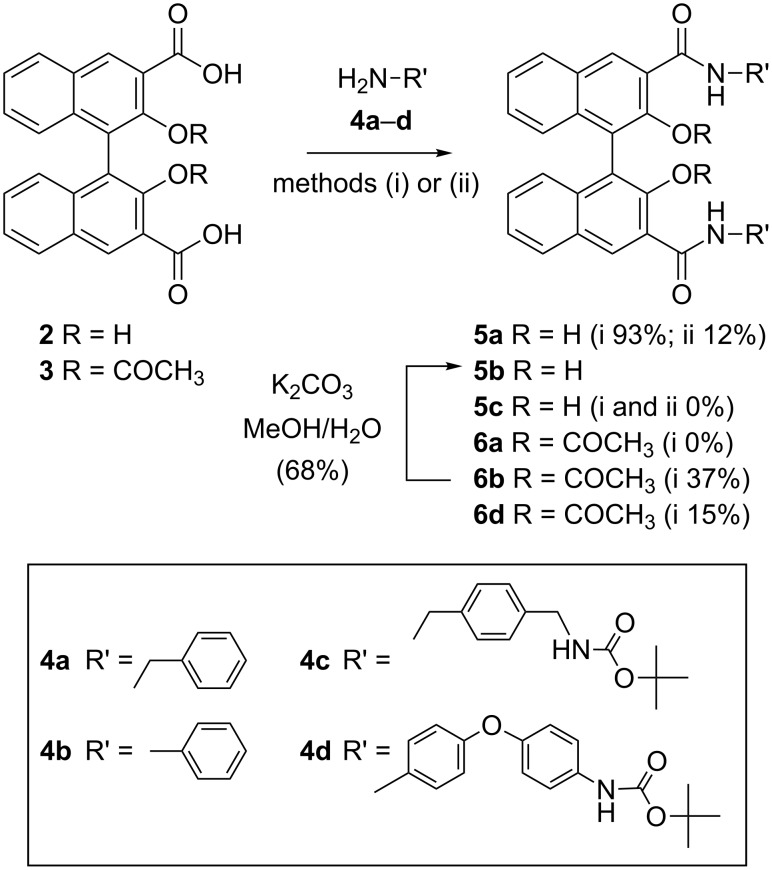
Reagents and conditions: (i) SOCl_2_, CHCl_3_ or (COCl)_2_, DMF, CH_2_Cl_2_ then amine, Et_3_N, CH_2_Cl_2_ or (ii) CDI, THF.

Preliminary synthetic work was performed on model compounds to test the reaction conditions. Both enantiomerically pure (*R*)-**2** and (*R*)-**3** and racemic (*RS*)-**2** and (*RS*)-**3** were used routinely in the experiments described in the following. Regarding approach (1), direct generation of the carboxylic acid chloride (with SOCl_2_, or oxalyl chloride and DMF; method i), followed by reaction with benzylamine (**4a**) in the presence of Et_3_N as the acid scavenger gave compound **5a** in excellent yields (93%) after purification by column chromatography (Scheme 1). The yield was higher in our hands than the one previously reported [35].

Alternative one-pot amidation procedures, performed directly with the aromatic carboxylic acid and the benzylic amine (method ii) using carbonyldiimidazole (CDI) [36], successfully used by us in the past [29], were instead less than satisfactory. Both protocols were applied with commercially available monoprotected benzylic diamine **4c**; in both cases, however, the desired product **5c** could not be isolated. It is likely that the BOC protecting group is not compatible with the presence of the free phenolic groups in our substrates.

Switching to approach (2), when compound **3** was allowed to react with benzylamine (**4a**) or aniline (**4b**), only the aryl derivate **6b** was obtained in good yield. Compound **6b** could be efficiently deprotected under the reported conditions (K_2_CO_3_/MeOH) to give **5b**. When the monoprotected aryl amine **4d** was allowed to react with compound **3** using the same reaction conditions, however, compound **6d** could not be efficiently synthesized. The low yield obtained in this step discouraged us from pursuing a stepwise methodology for the macrocyclization, which had been used in the case of (*R*,*R*)-**1** [31].

**Scheme 2:**
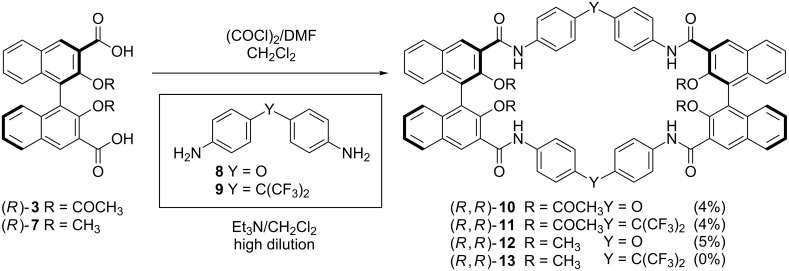
Structure and synthesis of the macrocycles discussed in this paper.

In order to quickly evaluate the potential of acetyl-protected tetraamidic macrocyles as analogues of (*R*,*R*)-**1**, we proceeded to directly cyclize equimolar amounts of optically pure, resolved (*R*)-**3** (via formation of the corresponding diacyl chloride) and commercially available diaryl amines **8** and **9**, under classical high-dilution conditions [13] which were successful for the synthesis of compound **6b**. Indeed, homochiral macrocycles (*R*,*R*)-**10**, (*R*,*R*)-**11** and (*R*,*R*)-**12** could be isolated after extensive purification by column chromatography, although in disappointingly low yields (0–5% isolated yield, Scheme 2).

Furthermore, macrocycle (*R*,*R*)-**13** could not be isolated at all. The low quantities of macrocycles (*R*,*R*)-**10** and (*R*,*R*)-**11** obtained prevented us from exploring the cleavage of the acetyl functionalities.

^1^H NMR spectra for all compounds were relatively simple (see Experimental and Supporting Information File 1), reflecting the structural symmetry found in precursors (*C*_2_ molecular symmetry) and in homochiral macrocycles **10**–**12** (*D*_2_ molecular symmetry). The peaks for the NH proton resonances of the amide functionalities are sharp in S(6)-type [37] hydrogen-bonded systems, such as those between the NH and the neighboring methoxy groups in (*R*,*R*)-**1** and (*R*,*R*)-**12**. The NH proton resonances, however, could not be assigned either in the series of compounds **5**, or in the acetoxy protected compounds **6** and macrocycles **10**,**11**, as they are broad or below the baseline, so as to indicate unlocked (thus potentially more available to incoming guests), conformationally mobile NH groups (Table 1).

**Table 1:** Selected chemical shifts for compounds in CDCl_3_ (25 °C).^a^

Entry	Compound	NH	Binol-H 4,4'^b^	OCH_3_	COCH_3_	OH

1	**5a**	9.88	8.74	—	—	12.44
2	**5b**	n. d.^c^	8.85	—	—	10.31
4	**6b**	n. d.	8.47	—	1.83	—
5	**6d**	n. d.	8.46	—	1.84	—
6	(*R*,*R*)-**10**	n. d.	8.58	—	1.82	—
7	(*R*,*R*)-**11**	n. d.	8.58	—	1.83	—
8	(*R*,*R*)-**12**	10.45	9.06	3.32	—	—
9	(*R*,*R*)-**1**	10.00	9.00	3.53	—	—

^a^All spectra recorded at 5–10 mM sample concentration. ^b^Resonances related to the singlet corresponding to the proton in the 4,4' positions of the binol skeleton. ^c^n. d. = broad or not identified.

There are also substantial differences in the resonances of the H 4,4' protons of the binaphthyl skeleton, which are usually most sensitive to variations in the substitution pattern (and thus, in the electronic structure) within the naphthyl systems of the binaphthyl units.

**Figure 2:**
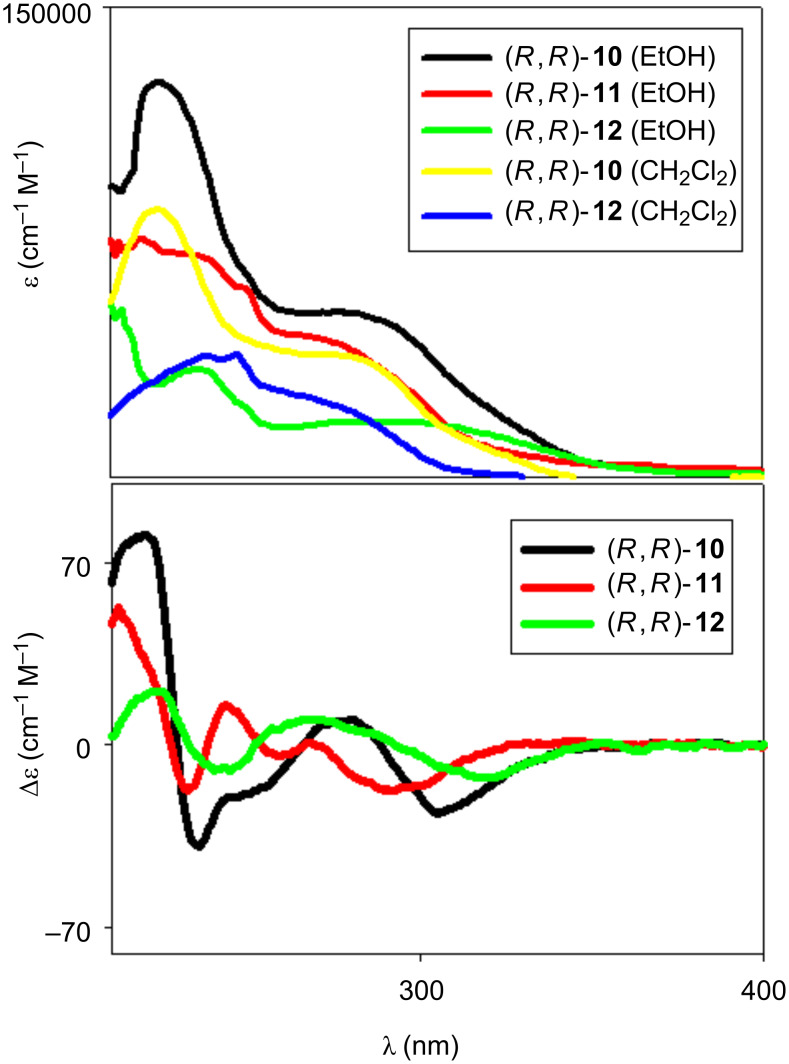
UV and CD (EtOH) spectra of macrocycles (*R*,*R*)-**10**, (*R*,*R*)-**11** and (*R*,*R*)-**12** in the range 220–400 nm.

The UV–vis spectra of macrocycles (*R*,*R*)-**10** and (*R*,*R*)-**11**, recorded in solvents possessing different solvating and hydrogen-bonding abilities (CH_2_Cl_2_, EtOH), showed little solvent dependence, with λ_max_ around 230 nm in all cases, and with well-defined shoulders just below 300 nm. Comparison with data available on parent systems [31] reveals that the spectra cannot be explained as the sum of those generated by the two major aromatic chromophoric components (the naphthyl rings of the binaphthyl units and the aryl moieties of the spacing units); electronic communication between them is present (Figure 2).

It is interesting to note how the spectra of macrocycle (*R*,*R*)-**12**, bearing methoxy protected phenols, showed the most bathochromically shifted shoulder, centered at 320 nm (quite different from (*R*,*R*)-**10**). The CD spectra (in EtOH) show activity associated with all active UV chromophores and more marked activity for the macrocycle (*R*,*R*)-**10**, with exciton couplet signals greater in intensity than the ones of the other macrocycles.

### Molecular modeling

**Figure 3:**
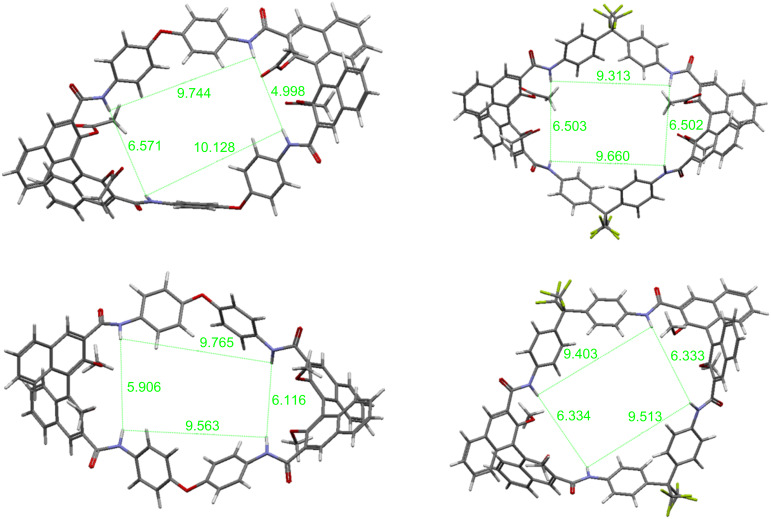
Minimized molecular structures of (from top left to bottom left, clockwise): (*R*,*R*)-**10**, (*R*,*R*)-**11**, (*R*,*R*)-**13** and (*R*,*R*)-**12** (distances between the H atoms of the NH amide groups are given in angstroms).

Molecular modeling was performed on the structures of macrocycles (*R*,*R*)*-***10**, (*R*,*R*)*-***11** and (*R*,*R*)*-***12**, and on the hypothetical macrocycle (*R*,*R*)*-***13**, in order to have an estimate of macrocyclic cavities, and to gather information on the relative orientation of the functional groups involved in the binding phenomena. Preliminary conformational structures were optimized by using the semiempirical PM3 method [38]. The geometries were then subjected to further refinement by using DFT B3LYP/6-31G(d) methods. In order to locate conformers having the minimum energy, the structures obtained by preliminary optimization were then subjected to molecular dynamics cycles and subsequent reoptimization [28]. The most stable minimized structures of the macrocycles are shown in Figure 3, in which the distances between the four hydrogen atoms of the NH amide groups within each macrocycle are reported (in angstroms).

The two macrocycles bearing aryloxy ether spacers ((*R*,*R*)*-***10** and (*R*,*R*)-**12**) have a rectangular overall geometry, probably as a consequence of the imposed dihedral angle of the oxygen atom bridges. In fact, the two trifluoromethyl-containing macrocycles ((*R*,*R*)-**11** and (*R*,*R*)*-***13**) have a more square-like geometry, which seems to be associated with the smaller imposed angle of the sp^3^-hybridized carbon atom in the spacing units. The dimensions of the macrocycles are essentially identical for the homologous sets of (*R*,*R*)*-***11** and (*R*,*R*)*-***13** (14 Å × 15.7 Å), and of (*R*,*R*)*-***10** and (*R*,*R*)*-***12** (12.5 Å × 16.2 Å). In the case of (*R*,*R*)*-***1**, the macrocycle cavity is more square-like, with the four amides all at a quite similar distance (ca. 6–7 Å). In the case of **10**–**13**, however, there is a substantial differentiation in distances between the two sets of amide functionalities, those linked to each different binaphthyl unit within the macrocycle. (*R*,*R*)*-***11** and (*R*,*R*)*-***13** possess slightly less distorted molecular conformations in which a *C*_2_ overall molecular symmetry seems to be retained. In fact, the dihedral angles of the binaphthyl units within the macrocycle are identical in the case of (*R*,*R*)*-***11** (76.1°) and of (*R*,*R*)*-***13** (79.5°), whereas they differ slightly in the case of the more distorted (*R*,*R*)*-***10** (70.8 and 72.6°) and (*R*,*R*)*-***12** (85.9 and 85.6°). Molecular modeling does not give clear hints as to why macrocycle (*R*,*R*)*-***13** could not be obtained.

The shortest distances between the symmetry-related NH groups, shown in Figure 3, are compatible with the insertion of either glutarate or succinate. In fact, the calculated dimensions of the two carboxylates are 5.3 Å for succinate and 6.5 Å for glutarate, considering their fully extended conformation. These data rationalize the preference shown in the case of macrocycle **12** for succinate; in detail, they suggest that the binding mode involves two NH groups linked to the same binaphthyl unit, rather than a complexation mode in which the bidentate guests are extended across the cavities of the macrocycles. These findings also explain why, in the presence of bulkier acetoxy groups on the binaphthyl units, this recognition mechanism in **10** and **11** is suppressed and alternative mechanisms take place (see below).

### Complexation studies

**Figure 4:**
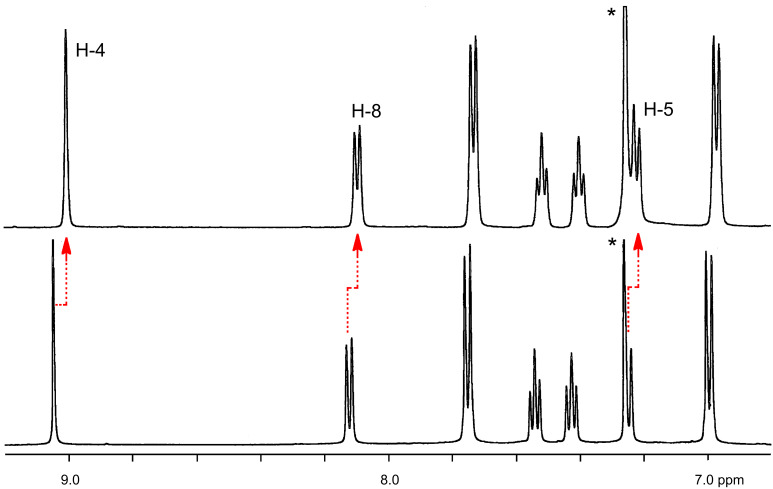
Aromatic region of the ^1^H NMR (CDCl_3_, 500 MHz, 25 °C) spectra of macrocycle (*R*,*R*)*-***12** (2.8 mM, bottom) and at the end of the titration with tetrabutylammonium succinate ([(*R*)-**7**] = 2.8 mM, [(CH_2_CO_2_)_2_(Bu_4_N)_2_] = 28 mM, top). Asterisks indicate the residual solvent peaks.

^1^H NMR titration experiments showed that the addition of anionic guests, in the form of their tetrabutylammonium salts, produced progressive chemical-shift variations, along with a broadening of the peaks belonging to the amide NH protons, indicating that these groups were engaged in hydrogen bonding with the carboxylate guests with a fast-exchanging equilibrium on the NMR time scale. Complexation-induced shifts on other resonances of the binaphthyl residues (Figure 4) indicated, as expected, a change of the electronic structure of these units upon complexation; these peaks were used for the calculation of the association constants, by using a 1:1 model equation (see Experimental).

Alternative binding models (i.e., stoichiometries) produced much poorer and hence unacceptable fitting of the titration data. In the case of (*R*,*R*)*-***12**, we examined dicarboxylate anions such as glutarate and succinate, in order to have a direct point of comparison with (*R*,*R*)*-***1** (Table 2).

**Table 2:** Binding constants (M^−1^) for the 1:1 complexes between (*R*,*R*)*-***12** or (*R*,*R*)*-***1** and dicarboxylate anions in CDCl_3_.^a^

Entry	Macrocycle	Succinate	Glutarate

1	(*R*,*R*)*-***12**	34 ± 3	21 ± 13
2^b^	(*R*,*R*)*-***1**	n. d.	30 ± 12

^a^Measured by ^1^H NMR (500 MHz, 298 K, CDCl_3_) using tetrabutylammonium salts. ^b^Data taken from Ref. [31]; n. d. = not determined.

Succinate was found to bind better than glutarate to macrocycle (*R*,*R*)*-***12**, probably as its length is more suited than glutarate to fit into the cavity of **12** by interacting simultaneously with two different NH groups placed on the same binaphthyl residue (see above, molecular modeling), possibly in a cooperative fashion [39].

Similar titrations were carried out on (*R*,*R*)*-***10** and (*R,R*)*-***11**, bearing acetoxy substituents in the 2,2**'** positions of the binaphthyl skeletons, which are certainly bulkier than the methoxy substituents present in (*R*,*R*)*-***12**. A very different behavior was observed for both macrocycles: The ^1^H NMR spectra in CDCl_3_, upon addition of both anionic guests, became very complex, with a complete loss of the initial symmetry. From the preliminary data in our hands, it is clear that there is a slow-exchanging equilibrium on the NMR time scale: the bulkier acetoxy group presumably inhibits a binding mechanism based on a fast host–guest exchange. A slow encapsulation mechanism, with multiple modes of binding of the difunctional anionic guests within the macrocycle*,* or the formation of aggregates of several macrocyclic units, held by the bifunctional anionic guests binding to a single amide of each macrocycle, cannot be ruled out at the present stage. Further studies are in progress to unravel the behavior of these acetoxy-bearing macrocycles.

## Conclusion

We have reported the synthesis and characterization of three novel homochiral macrocycles, built upon resolved 1,1'-binaphthyl scaffolds, which incorporate either methoxy or acetoxy functionalities in the 2,2' positions, and carboxylic functionalities in the external 3,3' positions. After evaluation of the synthetic strategy through test reactions on model compounds, the macrocycles were obtained through one-pot amidation reactions by using two different rigid diarylamines and high-dilution conditions, although in low isolated yields (0–5%). The macrocycle bearing less sterically demanding methoxy substituents is an effective supramolecular receptor for dicarboxylate anions, with a preference for glutarate (*K*_a_ = 34 M^−1^ for the 1:1 complex in CDCl_3_), as measured by ^1^H NMR spectroscopy. With the acetoxy groups installed within the macrocyclic framework, a drastic change of binding mode occurs, with slow aggregation equilibria on the NMR time scale.

## Experimental

**General.** All commercially available compounds were purchased from commercial sources and used as received. Racemic or (*R*)-**2** [40–41], racemic or (*R*)-**3** [42], **4c** [43], **4d** [44] and (*R*)-**7** [45] were prepared according to literature procedures. THF (Na), CH_2_Cl_2_ (CaH_2_) and CHCl_3_ (4 Å molecular sieves) were dried before use. Analytical thin-layer chromatography was performed on silica gel, chromophore-loaded, commercially available plates. Flash chromatography was carried out by using silica gel (pore size 60 Å, 230–400 mesh). ^1^H and ^13^C NMR spectra were recorded from solutions in CDCl_3_ on a 200, 300 or 500 MHz spectrometer with the solvent residual proton signal or tetramethylsilane as the standard. The UV–vis spectroscopic studies were recorded by using commercially available spectrophotometers. Mass spectra were recorded by using an electrospray ionization instrument. Optical rotations were measured on a polarimeter with a sodium lamp (λ = 589 nm) and are reported as follows: [α]_D_^rt^ (*c* = g (100 mL solution)^−1^). CD spectroscopy was performed by using a spectropolarimeter; spectra were recorded at 25 °C at a scanning speed of 50 nm min^−1^ and were background corrected.

**Compound 5a** [35]. SOCl_2_ (1.1 mL) was added to a solution of compound (*RS*)-**2** (250 mg, 0.67 mmol, 1 equiv) in dry CHCl_3_ (10 mL) and the solution was heated under reflux overnight. Then, the solution was concentrated in vacuo and the crude product was added to a solution of benzylamine cooled to 0 °C (430 mg, 4.00 mmol, 6 equiv) in dry CH_2_Cl_2_ (10 mL). After being stirred overnight at rt, the reaction mixture was quenched with 1 M HCl (30 mL), extracted with CH_2_Cl_2_ (3 × 25 mL), and dried (Na_2_SO_4_). The solution was filtered and concentrated in vacuo to yield pure **5a** (340 mg, 93%). ^1^H NMR (CDCl_3_, 300 MHz, 25 °C) δ 12.44 (s, 2H, OH), 9.88 (s, 2H, NH), 8.74 (s, 2H, binaphthyl), 7.91 (d, 2H, binaphthyl), 7.37 (m, 14H, phenyl + binaphthyl), 6.98 (d, 2H, binaphthyl), 4.60 (d, CH_2_). Alternative method: A solution of CDI (162 mg, 2.01 mmol, 3 equiv) in THF (12 mL) was added to a solution of (*R*)-**2** (250 mg, 0.668 mmol, 1 equiv) in THF (20 mL). The solution was stirred for 1.5 h at rt, and then a solution of benzylamine (0.146 mL, 1.34 mmol, 2 equiv) in THF (12 mL) was added and the mixture was stirred for 15 h. The reaction mixture was concentrated in vacuo, and the crude product was purified by column chromatography (hexane/AcOEt 9:1) to yield **5a** (44 mg, 12%).

**Compound 6b.** (COCl)_2_ (0.137 mL, 1.57 mmol, 8 equiv) and one drop of DMF were added to a solution of compound (*R*)-**3** (90 mg, 0.196 mmol, 1 equiv) and dry CH_2_Cl_2_ (10 mL). The solution was heated under reflux for 2 h. After 1 h at rt the solution was concentrated in vacuo, the crude product was dissolved in dry CH_2_Cl_2_ (5 mL), and a solution of **4b** (0.045 mL, 0.49 mmol, 2.5 equiv) and Et_3_N (0.082 mL, 0.59 mmol, 3 equiv) in dry CH_2_Cl_2_ (5 mL) was added. The resulting solution was heated under reflux for 2 h. After cooling, the reaction mixture was quenched with brine (20 mL), extracted with CH_2_Cl_2_ (3 × 25 mL), and dried (Na_2_SO_4_). The crude product was purified by column chromatography (hexane/AcOEt 8:2 to 7:3) to yield **6b** (44 mg, 37%). ^1^H NMR (CDCl_3_, 300 MHz, 25 °C) δ 8.47 (s, 2H, binaphthyl), 8.10 (s, 2H, binaphthyl), 8.03 (m, 2H, phenyl), 7.60 (m, 6H, binaphthyl + phenyl), 7.37 (m, 4H, binaphthyl), 7.22 (m, 4H, phenyl), 1.83 (s, 6H, –COCH_3_).

**Compound 5b.** K_2_CO_3_ (91 mg, 0.66 mmol, 8 equiv) and H_2_O (10 mL) were added to a solution of compound **6b** (44 mg, 0.082 mmol, 1 equiv) and CH_3_OH (10 mL). Then, the solution was stirred for 4 h at rt. The reaction mixture was quenched with 1 M HCl, extracted with CH_2_Cl_2_ and dried (Na_2_SO_4_). The solution was filtered and concentrated in vacuo and the crude product was purified by column chromatography (hexane/AcOEt 7:3) to yield **5b** (25 mg, 68%). ^1^H NMR (CDCl_3_, 300 MHz, 25 °C) δ 10.31 (brs, 2H, OH), 8.85 (s, 2H, binaphthyl), 7.97 (m, 2H, binaphthyl), 7.82 (m, 4H, phenyl), 7.40 (m, 8H, binaphthyl + phenyl), 7.17 (m, 4H, binaphthyl). The data are consistent with those reported in the literature [46].

**Compound 6d.** (COCl)_2_ (0.28 mL, 3.21 mmol, 8 equiv) and one drop of DMF were added to a solution of compound **3** (184 mg, 0.402 mmol, 1 equiv) and dry CH_2_Cl_2_ (15 mL). Then, the solution was heated at reflux for 2 h. After 1 h at rt the solution was concentrated in vacuo and the crude product was dissolved in dry CH_2_Cl_2_ (10 mL). This solution and a solution of **4d** (300 mg, 1 mmol, 2.5 equiv) in dry CH_2_Cl_2_ (10 mL) were added dropwise at the same rate over a period of 1 h to a solution of Et_3_N (0.279 mL, 2 mmol, 5 equiv) in dry CH_2_Cl_2_ (10 mL). The resulting solution was heated under reflux for 2 h. After cooling, the reaction mixture was quenched with brine (20 mL), extracted with CH_2_Cl_2_ (3 × 25 mL) and dried (Na_2_SO_4_). The crude product was purified by column chromatography (CH_2_Cl_2_/AcOEt 10:0 to 9:1) to yield **6d** (61 mg, 15%). ^1^H NMR (CDCl_3_, 300 MHz, 25 °C) δ 8.46 (s, 2H, binaphthyl), 8.04 (s, 4H, binaphthyl), 7.56 (d, 6H, binaphthyl + phenyl), 7.35 (m, 6H, binaphthyl + phenyl), 6.97 (d, 8H, phenyl), 1.84 (s, 6H, -COCH_3_), 1.53 (s, 18H, *t*-Bu).

**Macrocycle (*R*,*R*)*-*10.** (COCl)_2_ (0.381 mL, 4.38 mmol, 8 equiv) and one drop of DMF were added to a solution of compound (*R*)-**3** (250 mg, 0.546 mmol, 1 equiv) in dry CH_2_Cl_2_ (20 mL). The solution was heated under reflux for 2 h. After 1 h at rt the solvent was removed in vacuo and the crude product was dissolved in dry CH_2_Cl_2_ (35 mL). This solution and a solution of **8** (109 mg, 0.546 mmol, 1 equiv) in dry CH_2_Cl_2_ (35 mL) were added dropwise at the same rate over a period of 1 h to a solution of Et_3_N (0.228 mL, 1.64 mmol, 3 equiv) in dry CH_2_Cl_2_ (35 mL). The resulting solution was heated under reflux overnight. After cooling, the reaction mixture was quenched with brine (50 mL), extracted with CH_2_Cl_2_ (3 × 50 mL) and dried (Na_2_SO_4_). The solution was filtered and concentrated in vacuo, and the crude product was purified by column chromatography (CH_2_Cl_2_/AcOEt 9:1) to yield (*R*,*R*)*-***10** (7 mg, 4%). [α]_D_^25^ +101° (*c* 0.001, CH_2_Cl_2_); ^1^H NMR (CDCl_3_, 75 MHz, 25 °C) δ 8.58 (s, 4H, binaphthyl), 8.05 (m, 8H, phenyl), 7.50 (m, 16H, binaphthyl + phenyl), 6.97 (d, 8H, binaphthyl), 1.82 (s, 12H, -COCH_3_); ^13^C NMR (CDCl_3_, 75 MHz, 25 °C) δ 168.7 (O-CqOCH_3_), 163.5 (O-CqON), 154.6 (Cq), 143.1 (Cq), 133.8 (Cq), 133.3 (CH), 131.2 (Cq) 131.0 (Cq), 129.0 (CH), 128.7 (2CH), 128.0 (Cq), 127.0 (CH), 126.4 (CH), 125.1 (Cq), 121.1 (CH), 119.7 (2CH), 20.4 (COCH_3_); ESIMS *m*/*z*: 1267.5 ([M + Na]^+^, 100%).

**Macrocycle (*R,R*)*-*11.** The title compound was prepared by following the same procedure used for **10** but with diamine **9** used instead of **8**. The crude product was purified by column chromatography (CH_2_Cl_2_/AcOEt 98:2) to yield (*R*,*R*)*-***11** (18 mg, 4%). [α]_D_^25^ +56° (*c* 0.0015, CH_2_Cl_2_); ^1^H NMR (CDCl_3_, 75 MHz, 25 °C) δ 8.58 (s, 4H, binaphthyl), 8.25 (s, 4H, phenyl), 8.07 (d, 4H, phenyl), 7.40 (m, 24H, binaphthyl + phenyl), 1.83 (s, 12H, COCH_3_); ^13^C NMR (CDCl_3_, 75 MHz, 25 °C) δ 168.9 (O-CqOCH_3_), 163.8 (O-CqON), 143.0 (Cq), 138.1 (Cq), 133.9 (Cq), 131.3 (CH), 131.1 (2CH), 130.9 (Cq), 129.6 (Cq), 129.0 (CH), 128.9 (CH), 127.9 (Cq), 127.1 (CH), 126.3 (CH), 125.1 (Cq), 119.2 (2CH), 20.4 (CH_3_); ESIMS *m*/*z*: 1535.1 ([M + Na]^+^, 100%).

**Macrocycle (*R*,*R*)*-*12.** The title compound was prepared by following the same procedure used for **10** but with (*R*)-**7** used instead of (*R*)-**3**. The crude product was purified by column chromatography (CH_2_Cl_2_/AcOEt 99:1) to yield (*R*,*R*)*-***12** (6 mg, 5%). [α]^25^_D_ +170° (*c* 0.0015, CH_2_Cl_2_); ^1^H NMR (CDCl_3_, 300 MHz, 25 °C) δ 10.44 (s, 4H, NH), 9.07 (s, 4H, binaphthyl), 8.15 (d, 4H, binaphthyl), 7.78 (d, 8H, phenyl), 7.56 (t, 4H, binaphthyl), 7.45 (t, 4H, binaphthyl), 7.27 (d, 4H, binaphthyl), 7.02 (d, 4H, phenyl), 3.33 (s, 12H, O-CH_3_); ^13^C NMR (CDCl_3_, 75 MHz, 25 °C) δ 162.3 (O-CqON), 154.0 (Cq), 153.5 (Cq), 135.2 (Cq), 134.5 (CH), 133.9 (Cq), 130.4 (Cq), 129.9 (CH), 129.0 (CH), 126.0 (CH), 125.3 (CH+Cq), 124.9 (Cq), 121.0 (2CH), 119.3 (2CH), 62.0 (OCH_3_); ESIMS *m*/*z*: 1155.4 ([M + Na]^+^, 10%).

**^1^H NMR complexation experiments.** All spectra were recorded at 500 MHz and at 298 K. *K*_a_ values for the complexation of (*R*,*R*)*-***12** with (*n*-Bu_4_N^+^)_2_X^2−^ (X^2−^ = ^−^O_2_C(CH_2_)_2_CO_2_^−^, ^−^O_2_C(CH_2_)_3_CO_2_^−^) were assessed by nonlinear treatment of the data obtained from ^1^H NMR titration experiments. Samples were prepared by adding to a 0.5 mL solution of the host (5 mM in CDCl_3_) successive aliquots of a stock solution of the guest (62.5 mM in CDCl_3_), up to a final volume of 0.9 mL. Eight values of δ_obs_ for the H-4 resonances were collected by keeping the [host] to [guest] ratio in the (1:0.25)–(1:10) interval. Nonlinear regression analysis of δ_obs_ versus [guest], using the WinEQNMR for Windows software package [47], provided the *K*_a_ value.

**Molecular modeling.** Geometry optimizations for the structures presented were carried out, first by using the semiempirical PM3 method, and then refined at the B3LYP/6-31G(d) level [48]. All calculations were performed at the Cineca supercomputer facility by using the Gaussian 09, Revision C.01 package [49].

## Supporting Information

File 1 Additional NMR and MS spectra for the macrocyles, and Cartesian coordinates for the calculated geometries discussed in the paper.
