# Supplementary material for: Synthesis and anion recognition properties of shape-persistent binaphthyl-containing chiral macrocyclic amides
Source: Beilstein J Org Chem. 2012 Jun 28;8:967–76. doi: 10.3762/bjoc.8.109 (PMC3388887; doi:10.3762/bjoc.8.109)
Supplement: File 1 — Additional NMR and MS spectra for the macrocyles, and Cartesian coordinates for the calculated geometries discussed in the paper. [file Beilstein_J_Org_Chem-08-967-s001.pdf]

Supporting Information

for

Synthesis and anion recognition properties of shape-persistent binaphthyl-containing chiral macrocyclic amides

Marco Caricato[1], Nerea Jordana Leza[1], Claudia Gargiulli[2], Giuseppe Gattuso[2], Daniele Dondi[1] and Dario Pasini*[1,3]

Address: [1]Department of Chemistry, University of Pavia, Viale Taramelli 10, 27100 Pavia, Italy, [2]Department of Organic and Biological Chemistry, University of Messina, Viale F. Stagno d'Alcontres 31, 98166 Messina, Italy and [3]INSTM Research Unit, Department of Chemistry, University of Pavia, 27100 Pavia, Italy

Email: Dario Pasini* - dario.pasini@unipv.it

* Corresponding author

Additional NMR and MS spectra for the macrocyles, and Cartesian coordinates for the calculated geometries discussed in the paper.

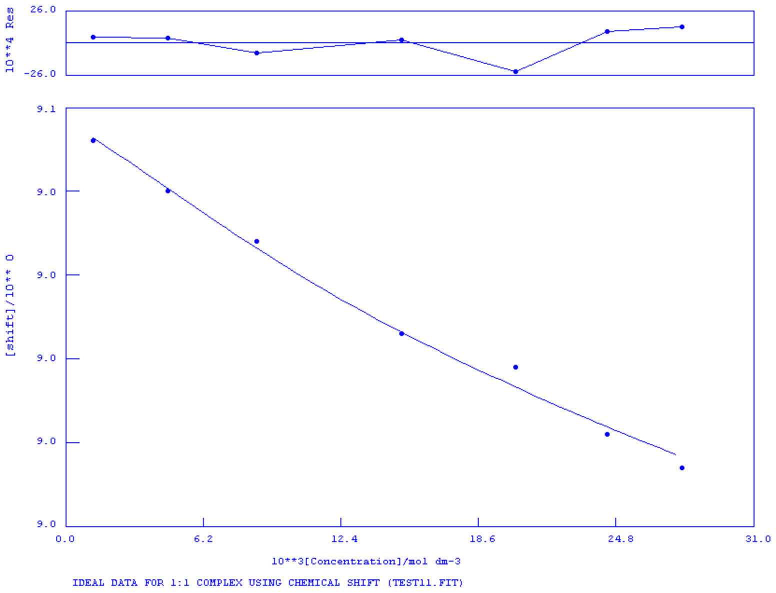

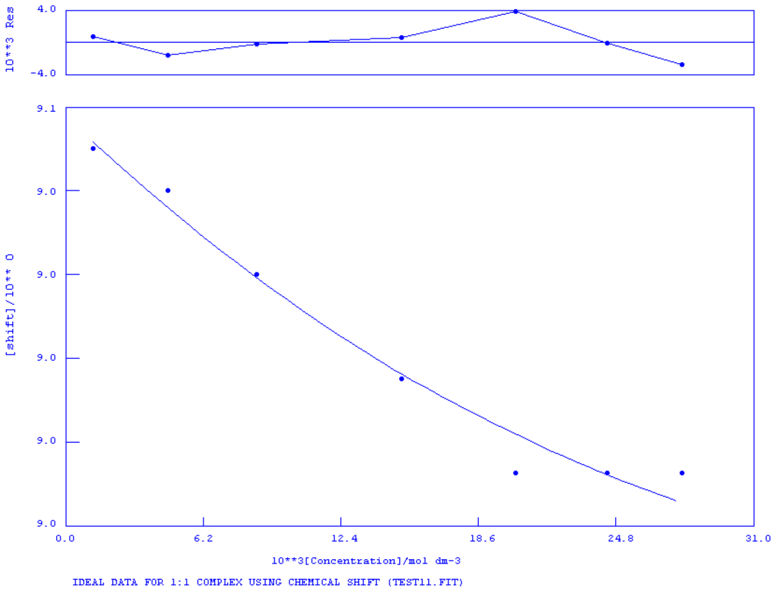

Binding profiles for the ^{1}H NMR titration experiments ($CDCl_3$, 500 MHz, 25 °C) carried out on receptor (*R*,*R*)-**12**; top: glutarate binding; bottom: succinate binding. See Experimental Section in the main manuscript for full details.

Compound (*R*,*S*)-**5a**.

^{1}H NMR ($CDCl_3$, 200 MHz, 25 °C).

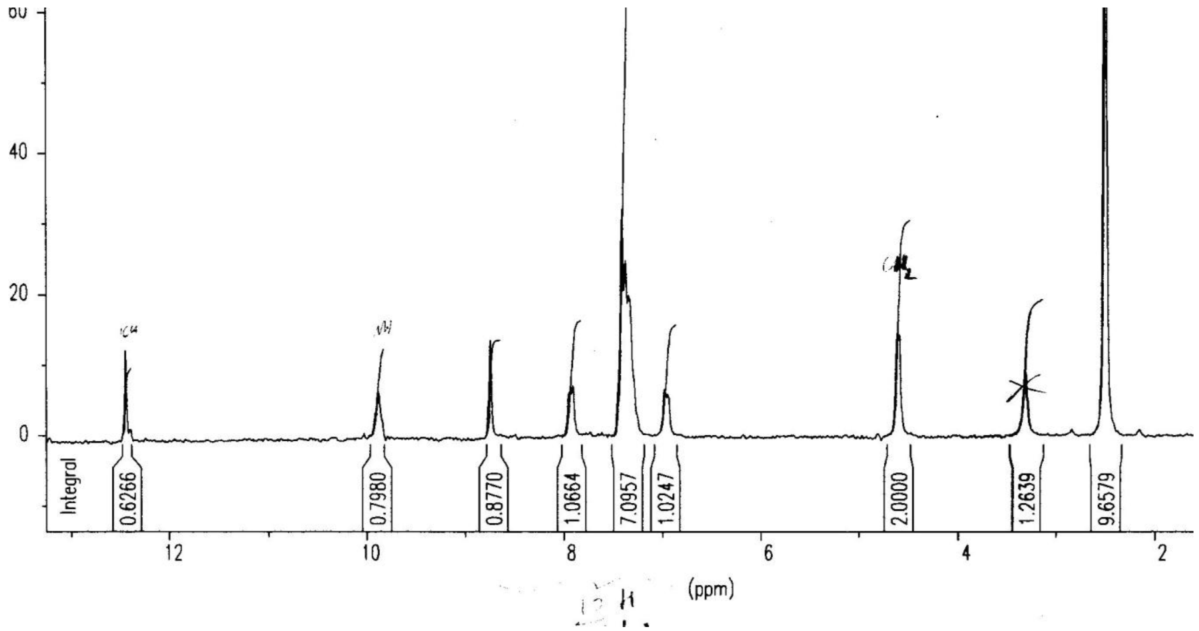

Compound (*R*,*S*)-**6b**.

^{1}H NMR ($CDCl_3$, 200 MHz, 25 °C).

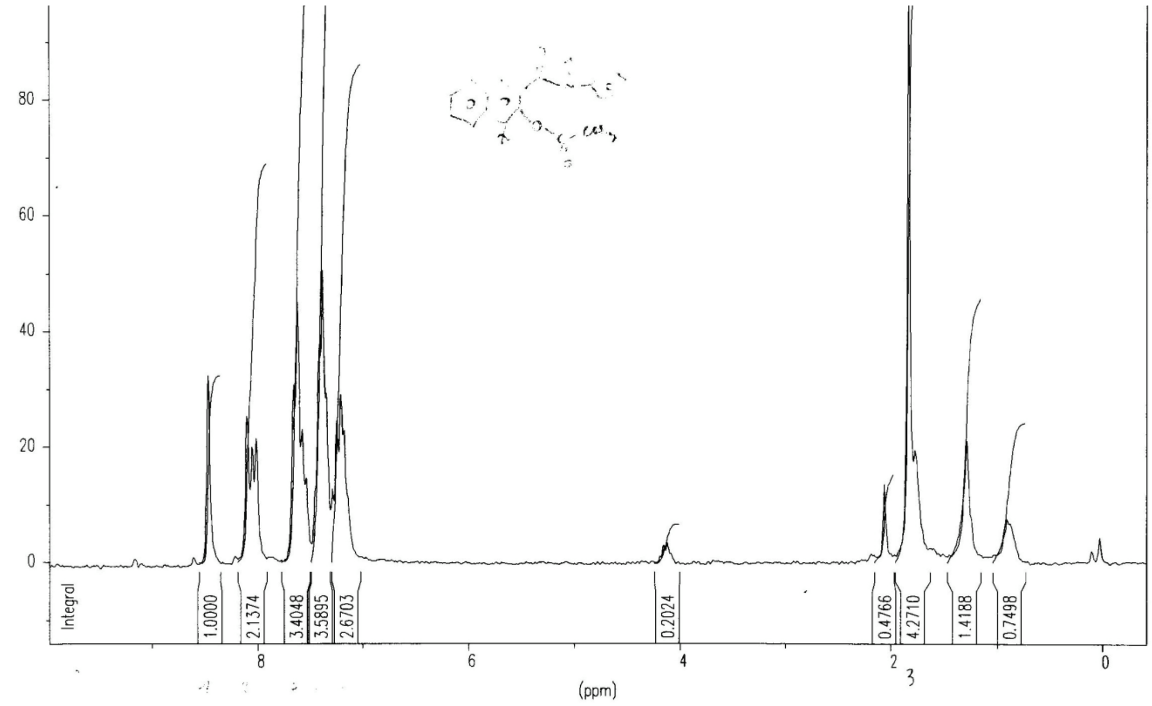

Compound (*R*,*S*)-**6d**.

^{1}H NMR ($CDCl_3$, 200 MHz, 25 °C).

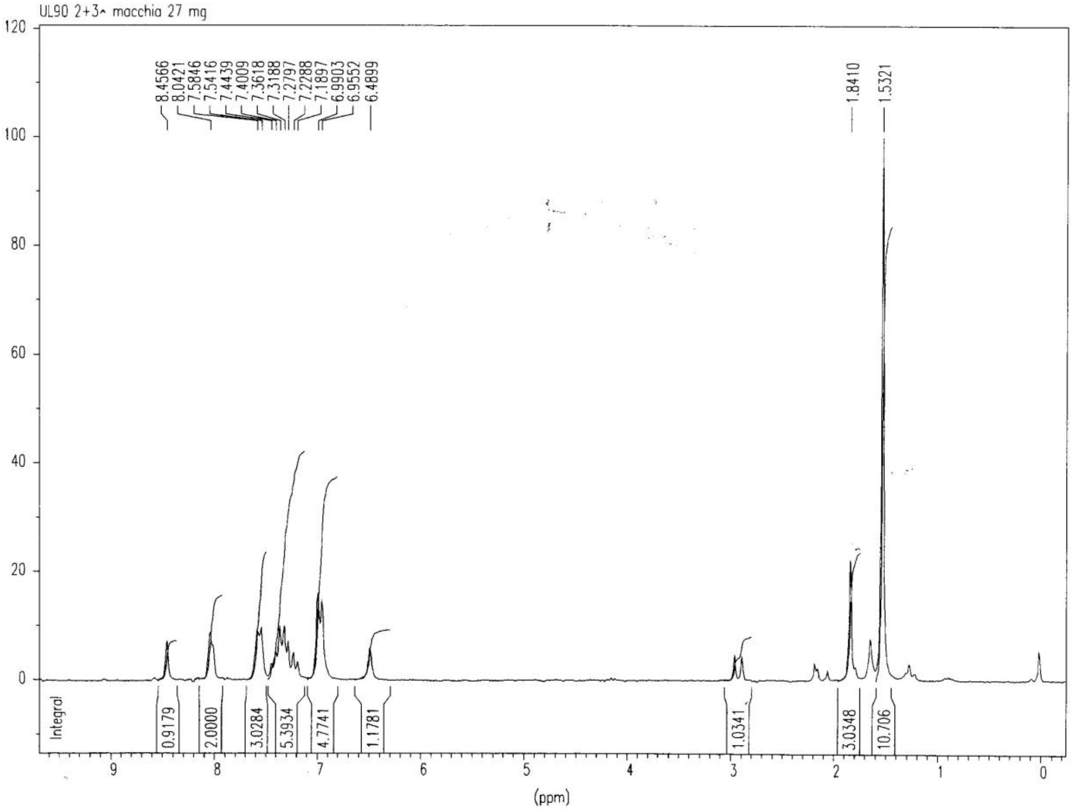

Macrocycle (*R*,*R*)-**10**.

^{1}H NMR ($CDCl_3$, 300 MHz, 25 °C).

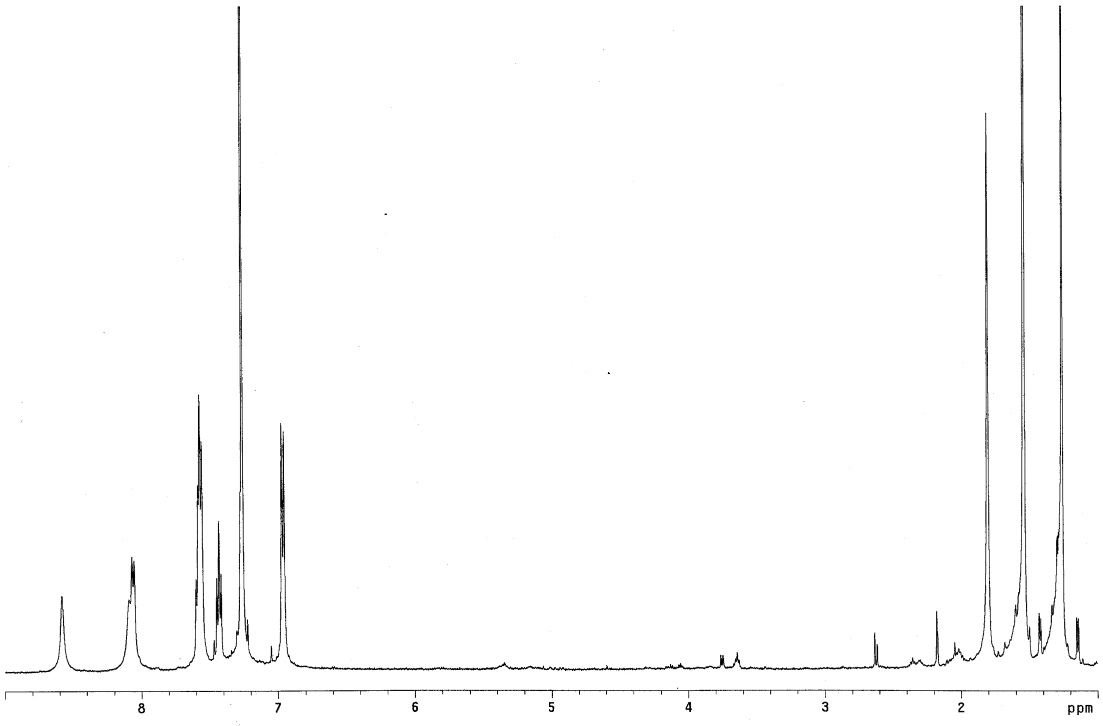

^{13}C NMR ($CDCl_3$, 75 MHz, 25 °C).

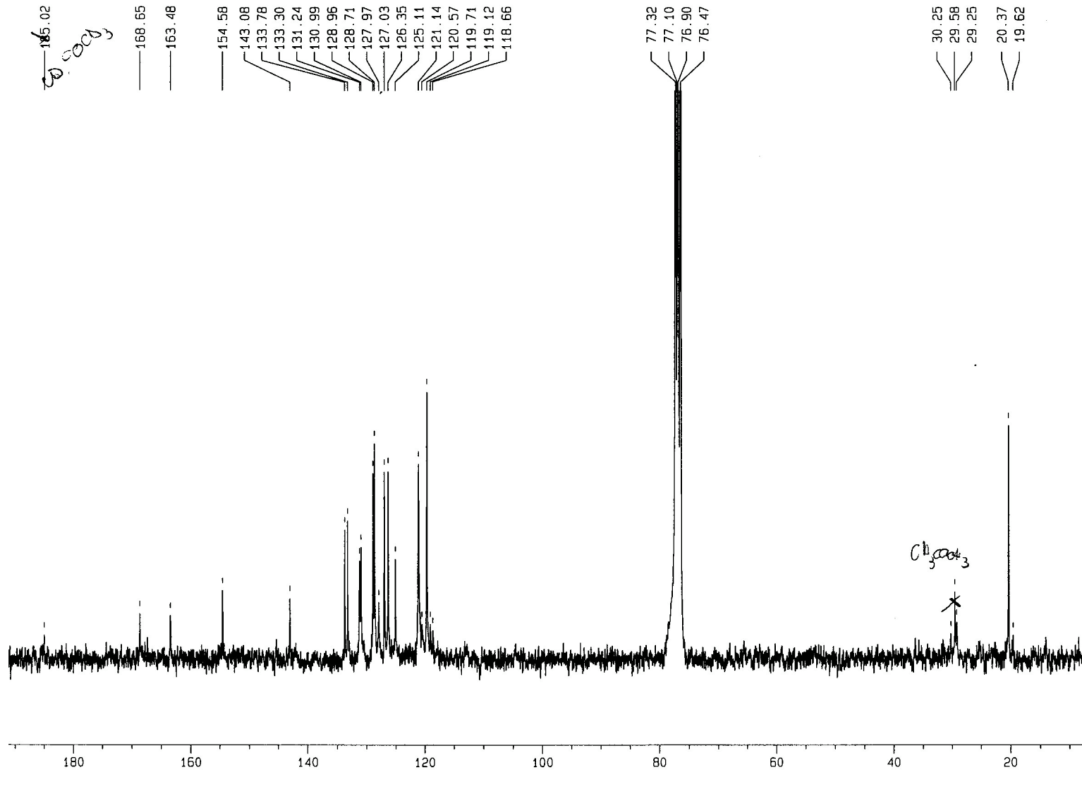

ESIMS.

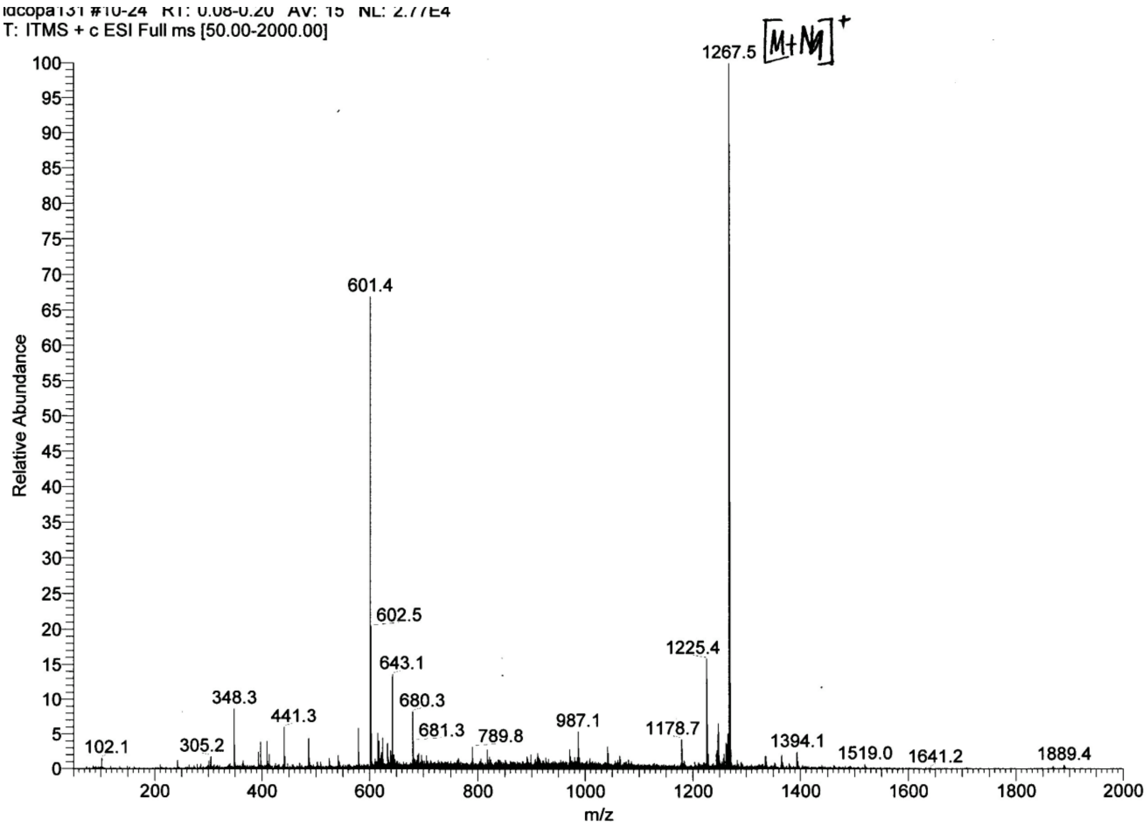
T: ITMS + c ESI Full ms [50.00-2000.00]
1267.5
[M+Na]+
601.4
602.5
643.1
680.3
681.3
789.8
987.1
1225.4
1178.7
1394.1
1519.0
1641.2
1889.4
348.3
441.3
305.2
102.1
Relative Abundance
m/z

Macrocycle (*R*,*R*)-**11**.

^{1}H NMR ($CDCl_3$, 300 MHz, 25 °C).

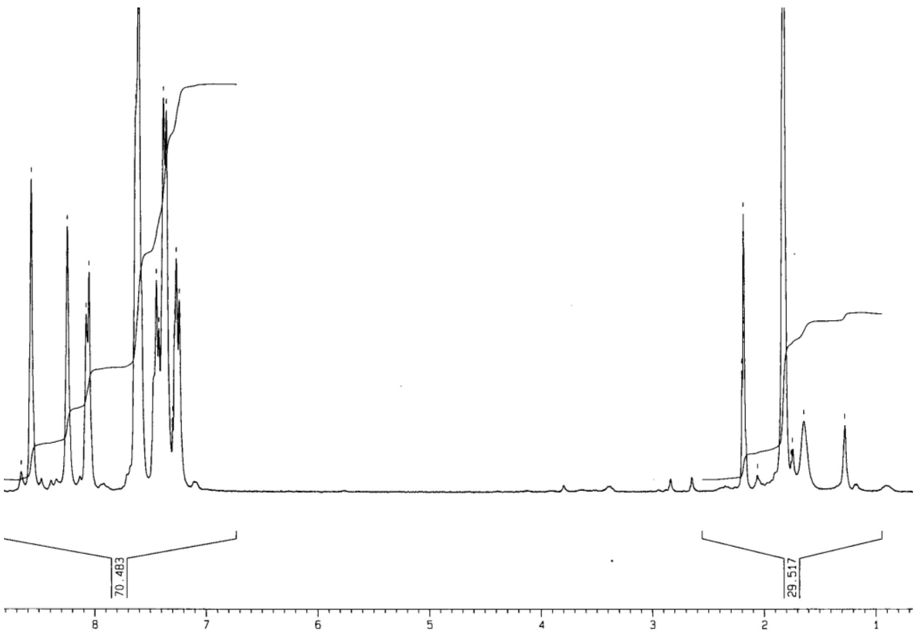

^{13}C NMR ($CDCl_3$, 75 MHz, 25 °C).

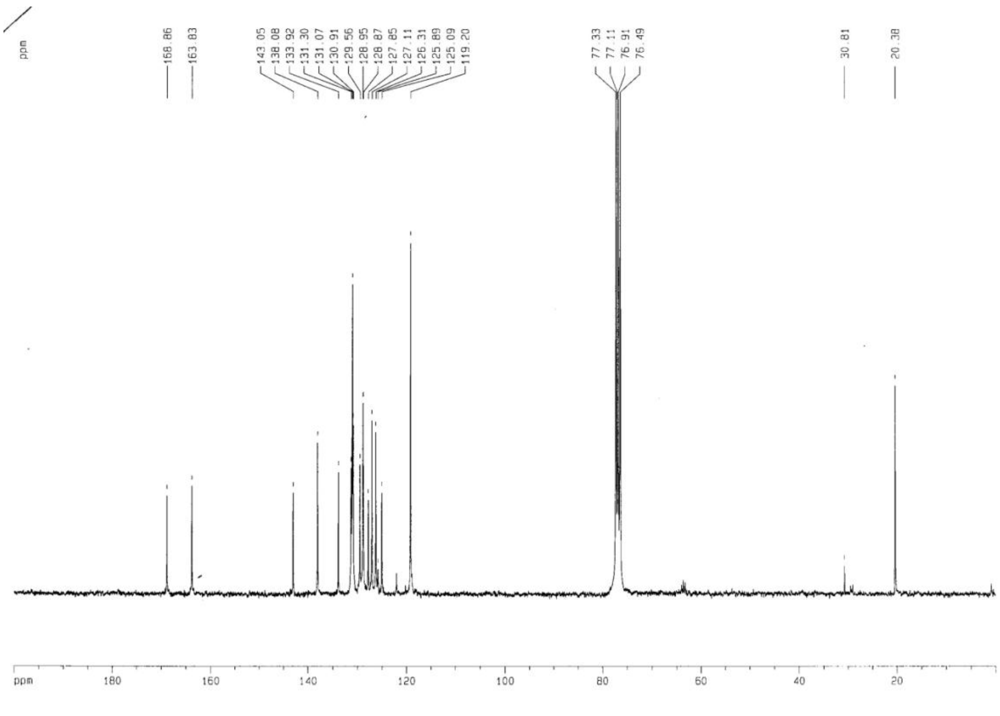

ESIMS.

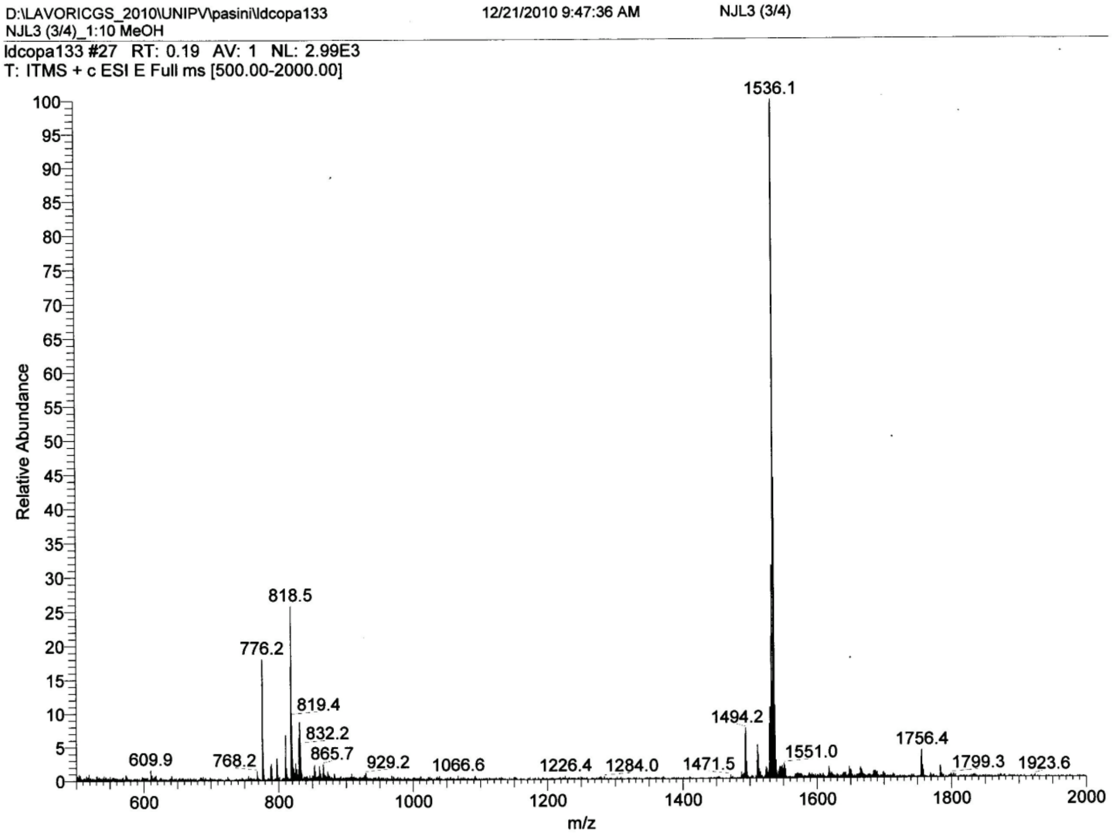
D:\LAVORICGS_2010\UNIPV\pasini\ldcopa133
NJL3 (3/4)_1:10 MeOH
12/21/2010 9:47:36 AM
NJL3 (3/4)
ldcopa133 #27 RT: 0.19 AV: 1 NL: 2.99E3
T: ITMS + c ESI E Full ms [500.00-2000.00]
1536.1
818.5
776.2
819.4
832.2
865.7
609.9
768.2
929.2
1066.6
1226.4
1284.0
1471.5
1494.2
1551.0
1756.4
1799.3
1923.6
Relative Abundance
m/z

Macrocycle (*R*,*R*)-**12**.

^{1}H NMR ($CDCl_3$, 300 MHz, 25 °C).

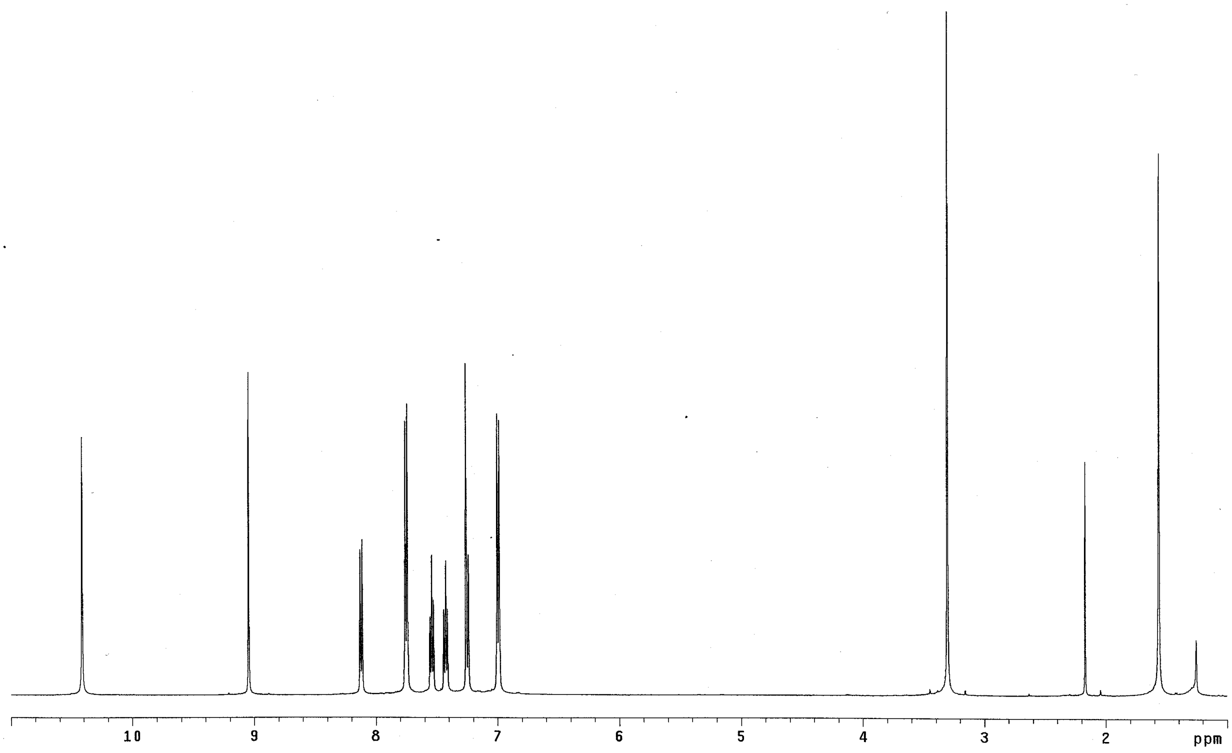

^{13}C NMR ($CDCl_3$, 75 MHz, 25 °C).

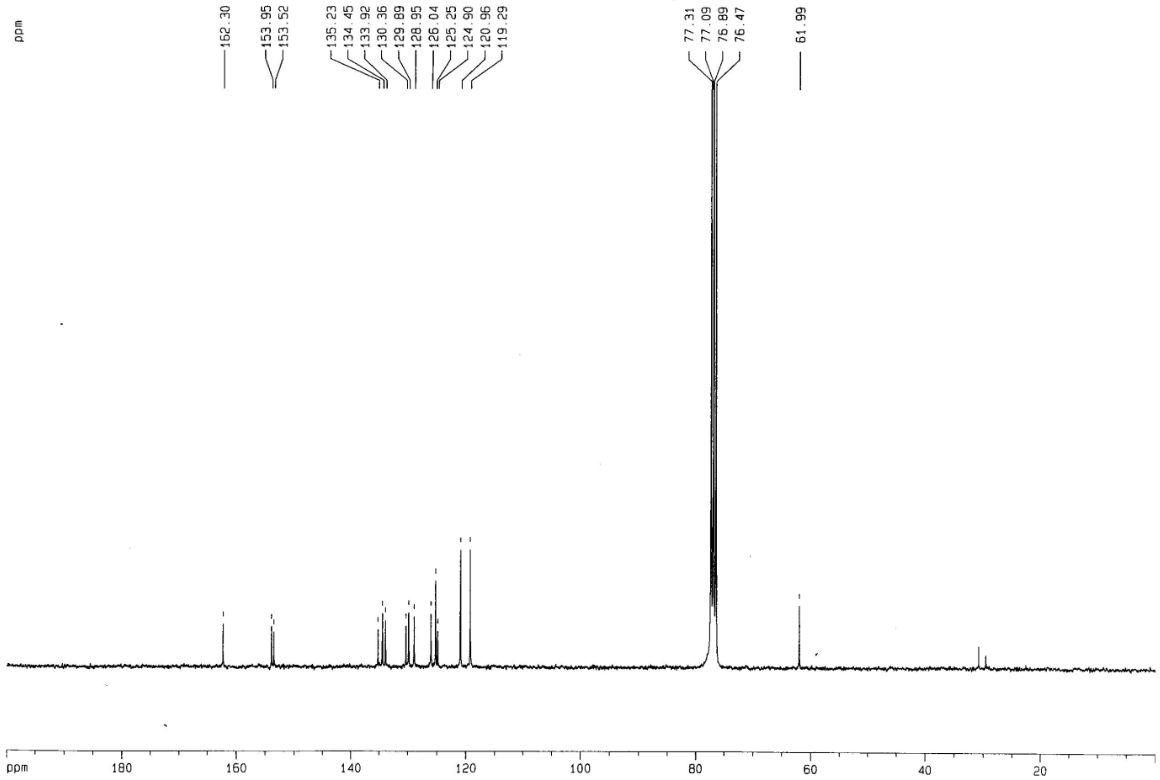

ESIMS.

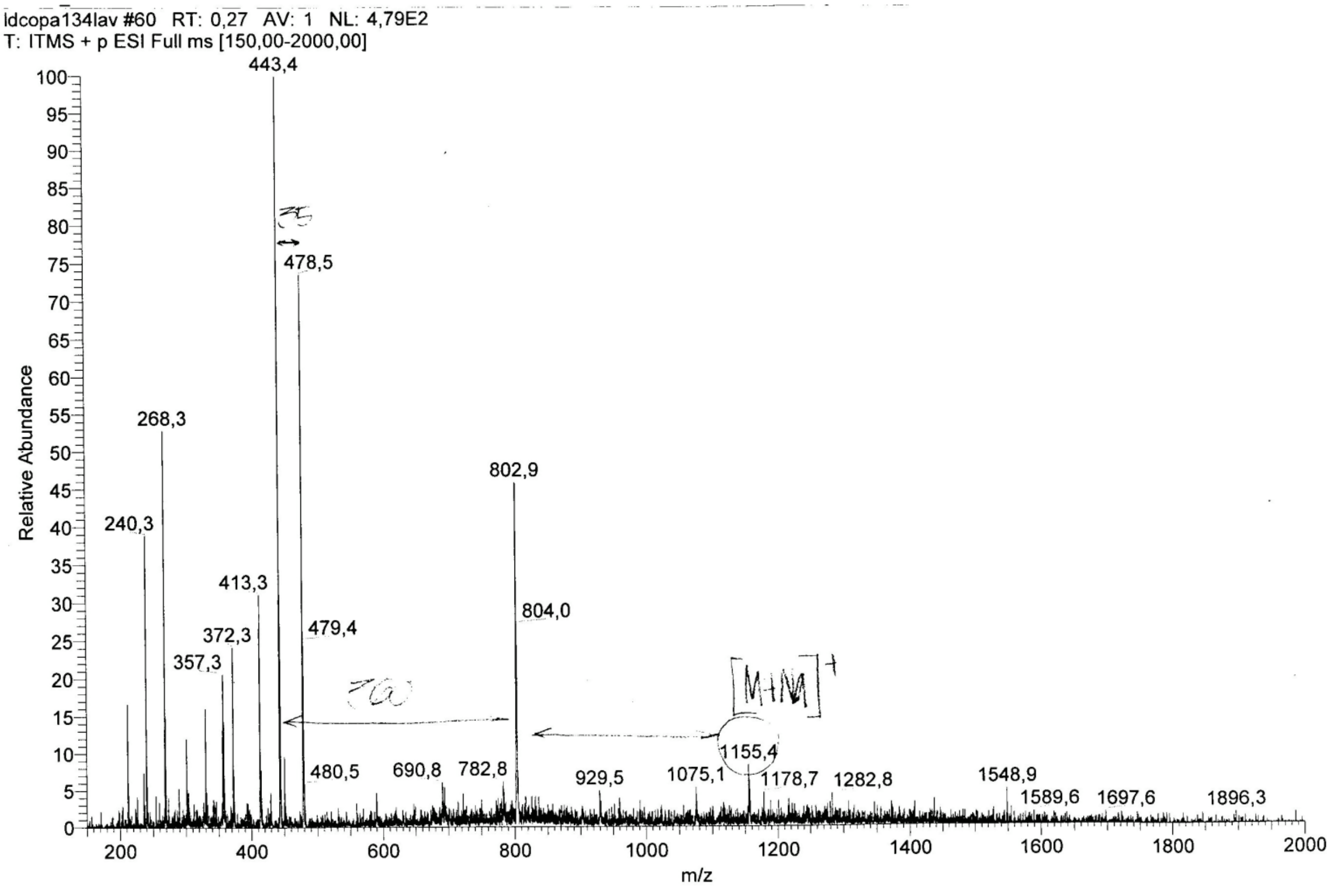

ldcopa134lav #60 RT: 0,27 AV: 1 NL: 4,79E2
T: ITMS + p ESI Full ms [150,00-2000,00]
443,4
478,5
268,3
240,3
802,9
413,3
804,0
372,3
479,4
357,3
[M+M]+
1155,4
480,5
690,8
782,8
929,5
1075,1
1178,7
1282,8
1548,9
1589,6
1697,6
1896,3
Relative Abundance
m/z

The Cartesian coordinates (in angstroms) for the calculated geometries reported in the article are listed below.

(R,R)-10

C	-11.05520000	0.00510000	2.91090000
C	-10.14080000	-0.23250000	1.90810000
C	-9.11610000	0.71080000	1.61480000
C	-9.06090000	1.91330000	2.39270000
C	-10.02340000	2.12820000	3.41690000
C	-11.00100000	1.19610000	3.67330000
H	-11.82870000	-0.72880000	3.11700000
C	-8.14300000	0.50540000	0.58100000
C	-8.04160000	2.85800000	2.13660000
H	-9.96660000	3.04560000	3.99590000
H	-11.73010000	1.36830000	4.45880000
C	-7.08000000	2.65530000	1.16730000
C	-7.16580000	1.46910000	0.38020000
H	-7.99240000	3.77620000	2.71260000
C	-8.16310000	-0.71950000	-0.28050000
C	-9.18940000	-0.91440000	-1.26490000
C	-7.15710000	-1.66670000	-0.16570000
C	-10.25060000	0.01170000	-1.46950000
C	-9.14840000	-2.08680000	-2.08740000
C	-7.06890000	-2.80680000	-1.01950000
C	-11.21670000	-0.21890000	-2.42460000
C	-10.16420000	-2.29600000	-3.06000000
C	-8.07970000	-2.99890000	-1.94000000
C	-11.17950000	-1.38400000	-3.22670000
H	-12.01790000	0.50110000	-2.56180000
H	-10.11670000	-3.19160000	-3.67290000
H	-8.02070000	-3.87890000	-2.57200000
H	-11.94990000	-1.55130000	-3.97280000
O	-6.13450000	-1.43800000	0.75650000
O	-6.17770000	1.21920000	-0.57120000
C	-6.04080000	3.75350000	1.03470000
O	-6.29770000	4.88430000	1.44170000
N	-4.84150000	3.38780000	0.48900000
H	-4.75050000	2.41650000	0.22080000
C	-5.95400000	-3.83900000	-1.06250000
O	-6.13380000	-4.90130000	-1.65290000
N	-4.77420000	-3.48770000	-0.46800000
H	-4.74190000	-2.57500000	-0.03320000
C	-3.56670000	-4.22340000	-0.42180000
C	-3.27840000	-5.30180000	-1.26990000
C	-2.60600000	-3.80390000	0.51200000
C	-2.02740000	-5.91270000	-1.20580000

H	-4.02560000	-5.64540000	-1.97050000
C	-1.36420000	-4.42480000	0.58160000
H	-2.84340000	-2.99560000	1.19890000
C	-1.06560000	-5.46970000	-0.29860000
H	-1.78270000	-6.73770000	-1.86640000
H	-0.63080000	-4.09830000	1.31030000
C	-3.70460000	4.18900000	0.23690000
C	-3.62560000	5.55890000	0.53210000
C	-2.60490000	3.55230000	-0.35750000
C	-2.46050000	6.26280000	0.23430000
H	-4.46840000	6.05450000	0.99160000
C	-1.44310000	4.25660000	-0.65120000
H	-2.66620000	2.49710000	-0.61060000
C	-1.37080000	5.61980000	-0.35320000
H	-2.38620000	7.32200000	0.45680000
H	-0.60390000	3.75170000	-1.11560000
O	0.13640000	-6.15320000	-0.26600000
O	-0.27180000	6.40030000	-0.67160000
C	1.33160000	-5.46670000	-0.17080000
C	2.41930000	-6.17170000	0.34890000
C	1.50690000	-4.15510000	-0.62010000
C	3.67640000	-5.57900000	0.44490000
H	2.26770000	-7.19370000	0.67980000
C	2.75670000	-3.55680000	-0.50860000
H	0.67730000	-3.60210000	-1.04370000
C	3.85190000	-4.25040000	0.02770000
H	4.51830000	-6.12560000	0.84530000
H	2.88070000	-2.52730000	-0.83650000
C	0.97850000	5.80420000	-0.67480000
C	1.55100000	5.33470000	0.50860000
C	1.68990000	5.73470000	-1.87160000
C	2.82430000	4.77260000	0.50450000
H	0.99200000	5.40580000	1.43570000
C	2.96450000	5.17380000	-1.88160000
H	1.23930000	6.11520000	-2.78180000
C	3.53560000	4.67510000	-0.70230000
H	3.27060000	4.40810000	1.41830000
H	3.51530000	5.11320000	-2.81730000
N	5.06470000	-3.53820000	0.12960000
C	6.29600000	-3.98420000	0.53120000
H	5.00200000	-2.55200000	-0.08220000
O	6.53830000	-5.14760000	0.83920000
N	4.79840000	4.04400000	-0.80460000
C	5.60320000	3.57450000	0.20450000
C	6.79960000	2.80060000	-0.28730000
C	7.66680000	3.32480000	-1.22290000
C	7.05120000	1.49900000	0.23750000
C	8.78300000	2.58810000	-1.69240000

H	7.50500000	4.32880000	-1.60770000
C	8.11630000	0.72630000	-0.20410000
C	9.67140000	3.12630000	-2.66190000
C	8.99920000	1.25860000	-1.20110000
C	10.71920000	2.37920000	-3.14670000
H	9.50270000	4.13910000	-3.01790000
C	10.07760000	0.50630000	-1.74210000
C	10.91430000	1.05460000	-2.68800000
H	11.39140000	2.79780000	-3.88940000
H	11.72930000	0.46190000	-3.09190000
C	7.36390000	-2.91620000	0.66830000
C	8.40590000	-3.21750000	1.52150000
C	7.36070000	-1.62890000	0.06250000
C	9.41710000	-2.27980000	1.83150000
H	8.42180000	-4.20590000	1.96900000
C	8.28850000	-0.64950000	0.36110000
C	10.47670000	-2.60690000	2.72110000
C	9.35300000	-0.96240000	1.26940000
C	11.43340000	-1.67660000	3.05210000
H	10.51120000	-3.60970000	3.13740000
C	10.35350000	-0.02210000	1.64110000
C	11.36570000	-0.37220000	2.50760000
H	12.23830000	-1.93680000	3.73250000
H	12.12110000	0.36070000	2.77490000
O	6.10720000	0.93740000	1.07570000
O	6.33880000	-1.31810000	-0.84700000
H	10.32110000	0.97840000	1.22490000
H	10.22090000	-0.51600000	-1.41410000
H	-10.30250000	0.90460000	-0.85700000
H	-10.20510000	-1.14390000	1.32440000
H	5.08840000	3.80540000	-1.74210000
O	5.38030000	3.74560000	1.39810000
C	-6.11240000	-2.00190000	2.03040000
O	-5.10030000	-1.86890000	2.66400000
C	-6.21050000	1.73320000	-1.86700000
O	-5.24620000	1.53460000	-2.55390000
C	6.12130000	1.05460000	2.46550000
O	5.23430000	0.51290000	3.06470000
C	6.61720000	-1.47170000	-2.19620000
O	7.69420000	-1.80320000	-2.61380000
C	-7.44720000	2.48620000	-2.28260000
H	-7.62600000	3.34010000	-1.62330000
H	-7.30020000	2.83540000	-3.30360000
H	-8.32710000	1.83910000	-2.23680000
C	-7.35110000	-2.71980000	2.49620000
H	-7.63340000	-3.51190000	1.79740000
H	-7.14310000	-3.14780000	3.47570000
H	-8.19150000	-2.02420000	2.56960000

C	7.25170000	1.83630000	3.08110000
H	8.22290000	1.43600000	2.78000000
H	7.18020000	2.87770000	2.75920000
H	7.15050000	1.77600000	4.16410000
C	5.38380000	-1.17940000	-3.00830000
H	4.65930000	-1.98940000	-2.87280000
H	4.91340000	-0.25230000	-2.67200000
H	5.65390000	-1.11340000	-4.06150000

(R,R)-11

C	10.23220000	0.32170000	-3.29880000
C	9.51240000	-0.02430000	-2.17700000
C	8.55250000	0.86740000	-1.62400000
C	8.33780000	2.12480000	-2.27820000
C	9.09900000	2.45120000	-3.43230000
C	10.03130000	1.57150000	-3.93150000
H	10.95780000	-0.37600000	-3.70700000
C	7.78650000	0.54290000	-0.45390000
C	7.35850000	3.01200000	-1.76450000
H	8.92960000	3.41130000	-3.91390000
H	10.60910000	1.82950000	-4.81440000
C	6.63000000	2.70050000	-0.63720000
C	6.85730000	1.45750000	0.02030000
H	7.19970000	3.96480000	-2.26440000
C	7.96050000	-0.76030000	0.26470000
C	9.09560000	-0.98740000	1.11040000
C	6.99890000	-1.74840000	0.15550000
C	10.14880000	-0.04260000	1.26170000
C	9.18350000	-2.22100000	1.83300000
C	7.04590000	-2.96110000	0.90000000
C	11.22520000	-0.30990000	2.07960000
C	10.30690000	-2.46340000	2.67020000
C	8.14230000	-3.16700000	1.71460000
C	11.30930000	-1.53010000	2.79210000
H	12.02030000	0.42420000	2.17720000
H	10.35520000	-3.40430000	3.21190000
H	8.18620000	-4.09640000	2.27300000
H	12.16470000	-1.72410000	3.43260000
O	5.91440000	-1.52090000	-0.70830000
O	5.99740000	1.10130000	1.04410000
C	5.65780000	3.69730000	-0.05730000
O	5.82590000	4.20380000	1.04470000
N	4.60490000	3.99940000	-0.88930000
H	4.51560000	3.41800000	-1.71230000
C	6.00980000	-4.06680000	0.93640000
O	6.28730000	-5.14770000	1.44450000
N	4.76810000	-3.77060000	0.42570000
H	4.65660000	-2.84410000	0.03390000

C	3.63810000	-4.60980000	0.36820000
C	3.50380000	-5.79030000	1.11150000
C	2.58220000	-4.22400000	-0.47180000
C	2.33880000	-6.54720000	1.01240000
H	4.31120000	-6.11430000	1.75160000
C	1.42910000	-4.99350000	-0.56810000
H	2.66290000	-3.31190000	-1.05900000
C	1.27230000	-6.16470000	0.18750000
H	2.28200000	-7.45350000	1.60020000
H	0.64060000	-4.67110000	-1.23850000
C	3.51170000	4.85180000	-0.61600000
C	3.57460000	5.89920000	0.31000000
C	2.32060000	4.64140000	-1.32370000
C	2.46240000	6.71500000	0.51420000
H	4.48200000	6.06910000	0.87190000
C	1.21330000	5.44760000	-1.09620000
H	2.25200000	3.82880000	-2.04370000
C	1.25930000	6.50020000	-0.16870000
H	2.56010000	7.52450000	1.22490000
H	0.29950000	5.24740000	-1.64360000
C	-1.27150000	-6.16490000	-0.18450000
C	-2.33790000	-6.54840000	-1.00900000
C	-1.42830000	-4.99290000	0.56980000
C	-3.50290000	-5.79170000	-1.10900000
H	-2.28100000	-7.45530000	-1.59590000
C	-2.58150000	-4.22360000	0.47260000
H	-0.63980000	-4.66970000	1.23990000
C	-3.63740000	-4.61030000	-0.36700000
H	-4.31030000	-6.11640000	-1.74880000
H	-2.66220000	-3.31080000	1.05880000
C	-1.26030000	6.50010000	0.17150000
C	-2.46340000	6.71530000	-0.51130000
C	-1.21450000	5.44700000	1.09840000
C	-3.57560000	5.89940000	-0.30790000
H	-2.56090000	7.52530000	-1.22160000
C	-2.32180000	4.64060000	1.32520000
H	-0.30080000	5.24640000	1.64590000
C	-3.51280000	4.85140000	0.61740000
H	-4.48290000	6.06970000	-0.86980000
H	-2.25330000	3.82760000	2.04470000
N	-4.76750000	-3.77140000	-0.42530000
C	-6.00870000	-4.06750000	-0.93720000
H	-4.65630000	-2.84480000	-0.03360000
O	-6.28570000	-5.14830000	-1.44570000
N	-4.60610000	3.99880000	0.88990000
C	-5.65860000	3.69710000	0.05740000
C	-6.63100000	2.70000000	0.63630000
C	-7.36090000	3.01140000	1.76280000

C	-6.85730000	1.45690000	-0.02140000
C	-8.34050000	2.12380000	2.27540000
H	-7.20290000	3.96420000	2.26290000
C	-7.78680000	0.54210000	0.45160000
C	-9.10310000	2.45000000	3.42870000
C	-8.55420000	0.86640000	1.62090000
C	-10.03570000	1.57000000	3.92680000
H	-8.93450000	3.41010000	3.91050000
C	-9.51450000	-0.02560000	2.17280000
C	-10.23550000	0.32020000	3.29390000
H	-10.61450000	1.82780000	4.80910000
H	-10.96140000	-0.37770000	3.70130000
C	-7.04470000	-2.96180000	-0.90180000
C	-8.14030000	-3.16760000	-1.71760000
C	-6.99850000	-1.74910000	-0.15730000
C	-9.18140000	-2.22150000	-1.83700000
H	-8.18360000	-4.09700000	-2.27610000
C	-7.96000000	-0.76100000	-0.26730000
C	-10.30390000	-2.46390000	-2.67540000
C	-9.09420000	-0.98800000	-1.11420000
C	-11.30620000	-1.53070000	-2.79820000
H	-10.35170000	-3.40480000	-3.21720000
C	-10.14730000	-0.04330000	-1.26650000
C	-11.22280000	-0.31040000	-2.08550000
H	-12.16090000	-1.72460000	-3.43960000
H	-12.01790000	0.42360000	-2.18380000
O	-5.99600000	1.10110000	-1.04420000
O	-5.91480000	-1.52170000	0.70760000
H	-10.10480000	0.89280000	-0.72000000
H	-9.65930000	-0.99510000	1.71130000
H	10.10580000	0.89350000	0.71540000
H	9.65800000	-0.99380000	-1.71570000
H	-4.51710000	3.41710000	1.71280000
O	-5.82640000	4.20420000	-1.04440000
C	-0.00050000	7.37930000	0.00170000
C	0.00040000	-7.02420000	0.00190000
C	-0.07610000	8.30130000	-1.25260000
C	0.07510000	8.30060000	1.25670000
C	-0.27220000	-7.93770000	1.23480000
C	0.27320000	-7.93890000	-1.23010000
F	-1.09120000	8.93140000	1.47170000
F	1.02800000	9.25270000	1.14920000
F	0.35330000	7.58590000	2.36750000
F	1.09020000	8.93220000	-1.46730000
F	-0.35430000	7.58730000	-2.36380000
F	-1.02890000	9.25340000	-1.14450000
F	0.25660000	-7.21490000	-2.36720000
F	1.47480000	-8.53440000	-1.14840000

F	-0.63950000	-8.92890000	-1.36710000
F	0.64050000	-8.92760000	1.37290000
F	-1.47390000	-8.53330000	1.15370000
F	-0.25580000	-7.21260000	2.37130000
C	-6.20820000	1.36620000	-2.39830000
O	-5.36400000	0.98610000	-3.16140000
C	-6.11270000	-1.79900000	2.05360000
O	-7.14210000	-2.23560000	2.49220000
C	6.11100000	-1.79800000	-2.05450000
O	7.14000000	-2.23480000	-2.49400000
C	6.21110000	1.36680000	2.39790000
O	5.36770000	0.98680000	3.16210000
C	4.85600000	-1.50310000	-2.83460000
H	5.11360000	-1.36020000	-3.88500000
H	4.17600000	-2.36050000	-2.76140000
H	4.34130000	-0.62450000	-2.43870000
C	7.47120000	2.10310000	2.77020000
H	7.49210000	2.20410000	3.85590000
H	8.36080000	1.56100000	2.43790000
H	7.46370000	3.09260000	2.30500000
C	-4.85830000	-1.50440000	2.83480000
H	-5.11640000	-1.36350000	3.88530000
H	-4.17750000	-2.36100000	2.76030000
H	-4.34420000	-0.62470000	2.44060000
C	-7.46800000	2.10220000	-2.77210000
H	-7.48760000	2.20320000	-3.85780000
H	-8.35790000	1.56000000	-2.44080000
H	-7.46120000	3.09180000	-2.30690000

(R,R)-12

C	10.81010000	-0.47830000	-2.65880000
C	9.88630000	-0.57500000	-1.65380000
C	8.99250000	0.49860000	-1.38470000
C	9.07140000	1.66120000	-2.18000000
C	10.04130000	1.73860000	-3.21640000
C	10.89080000	0.69160000	-3.44870000
H	11.49800000	-1.30640000	-2.86080000
C	8.02310000	0.42860000	-0.33600000
C	8.17830000	2.74140000	-1.94910000
H	10.09470000	2.65010000	-3.82320000
H	11.63980000	0.74710000	-4.24560000
C	7.24170000	2.66320000	-0.95250000
C	7.17560000	1.50270000	-0.12430000
H	8.24930000	3.63450000	-2.58280000
C	7.92390000	-0.78580000	0.49780000
C	8.77090000	-0.93030000	1.64150000
C	7.00070000	-1.77580000	0.20950000
C	9.74190000	0.05060000	1.98610000

C	8.64770000	-2.07600000	2.45490000
C	6.84330000	-2.90570000	1.06820000
C	10.54950000	-0.12090000	3.07760000
C	9.50030000	-2.23320000	3.58130000
C	7.66370000	-3.05560000	2.15580000
C	10.43050000	-1.27640000	3.88350000
H	11.29760000	0.63570000	3.33770000
H	9.39780000	-3.13220000	4.20030000
H	7.56620000	-3.92950000	2.81270000
H	11.09010000	-1.39350000	4.74980000
O	6.12470000	-1.64340000	-0.86790000
O	6.16080000	1.43930000	0.82890000
C	6.28510000	3.79640000	-0.73340000
O	6.54380000	4.77360000	-0.05260000
N	5.06170000	3.68380000	-1.46460000
H	4.88250000	2.74890000	-1.76470000
C	5.76970000	-3.91970000	0.81790000
O	5.98640000	-5.07770000	0.50430000
N	4.44640000	-3.45040000	1.08440000
H	4.34880000	-2.46520000	0.95800000
C	3.27620000	-4.17230000	0.63240000
C	2.94590000	-5.40010000	1.21650000
C	2.43130000	-3.59440000	-0.32290000
C	1.75290000	-6.02840000	0.88140000
H	3.62250000	-5.87300000	1.93830000
C	1.24430000	-4.22260000	-0.67150000
H	2.69190000	-2.64170000	-0.80090000
C	0.89710000	-5.42690000	-0.04940000
H	1.48680000	-6.98680000	1.34240000
H	0.58130000	-3.77300000	-1.42030000
C	3.84920000	4.35910000	-1.06040000
C	3.81820000	5.74720000	-0.87660000
C	2.67240000	3.61020000	-0.93330000
C	2.62520000	6.37910000	-0.55350000
H	4.73270000	6.34540000	-0.97250000
C	1.47410000	4.23640000	-0.62200000
H	2.67700000	2.52260000	-1.08030000
C	1.45290000	5.62070000	-0.42680000
H	2.60360000	7.46470000	-0.40230000
H	0.55570000	3.64430000	-0.52660000
O	-0.24450000	-6.15150000	-0.36950000
O	0.32050000	6.35990000	-0.12060000
C	-1.40920000	-5.42860000	-0.58400000
C	-2.34530000	-6.04990000	-1.42120000
C	-1.69830000	-4.20690000	0.03370000
C	-3.56240000	-5.42600000	-1.66540000
H	-2.12230000	-7.02020000	-1.88020000
C	-2.91070000	-3.58380000	-0.22500000

H	-0.97270000	-3.73900000	0.71000000
C	-3.83720000	-4.18350000	-1.08520000
H	-4.30080000	-5.91260000	-2.31360000
H	-3.12810000	-2.61790000	0.24830000
C	-0.84400000	5.62230000	0.09540000
C	-1.84640000	5.67040000	-0.87760000
C	-1.04490000	4.94720000	1.30400000
C	-3.04720000	5.00680000	-0.65690000
H	-1.68680000	6.22280000	-1.81090000
C	-2.24540000	4.28670000	1.52000000
H	-0.26040000	4.93420000	2.06920000
C	-3.24520000	4.29860000	0.53490000
H	-3.82810000	5.04400000	-1.42660000
H	-2.39160000	3.74840000	2.46490000
N	-5.05250000	-3.47680000	-1.44180000
C	-6.30820000	-3.83200000	-0.85530000
H	-4.90270000	-2.49020000	-1.46450000
O	-6.51510000	-4.95230000	-0.42280000
N	-4.41020000	3.48140000	0.73580000
C	-5.74740000	3.84870000	0.42290000
C	-6.76870000	2.84840000	0.87310000
C	-7.44670000	3.05130000	2.04660000
C	-7.02860000	1.68290000	0.09150000
C	-8.38570000	2.09130000	2.50830000
H	-7.27420000	3.95440000	2.64540000
C	-7.91220000	0.71310000	0.53580000
C	-9.09240000	2.30150000	3.72390000
C	-8.61040000	0.91220000	1.76670000
C	-9.97840000	1.36420000	4.18000000
H	-8.91460000	3.22600000	4.28570000
C	-9.53210000	-0.04720000	2.27100000
C	-10.19760000	0.17580000	3.44570000
H	-10.52580000	1.52250000	5.11510000
H	-10.90880000	-0.56390000	3.82890000
C	-7.34450000	-2.75210000	-0.92140000
C	-8.38870000	-2.86350000	-1.80130000
C	-7.22830000	-1.58990000	-0.10010000
C	-9.33620000	-1.81240000	-1.92820000
H	-8.50100000	-3.75990000	-2.42470000
C	-8.10820000	-0.53030000	-0.23580000
C	-10.42260000	-1.92900000	-2.83730000
C	-9.19030000	-0.63510000	-1.16460000
C	-11.31760000	-0.90400000	-2.98110000
H	-10.52820000	-2.85240000	-3.41880000
C	-10.13130000	0.41650000	-1.34290000
C	-11.16720000	0.28250000	-2.22730000
H	-12.15650000	-0.99000000	-3.67970000
H	-11.89130000	1.09350000	-2.35950000

O	-6.28600000	1.49070000	-1.07150000
O	-6.13760000	-1.50340000	0.76550000
H	-10.01290000	1.33420000	-0.75290000
H	-9.70300000	-0.96550000	1.69480000
H	9.83420000	0.94430000	1.35580000
H	9.82430000	-1.47920000	-1.03470000
C	6.63640000	-2.18270000	-2.07070000
H	7.58100000	-1.70520000	-2.36200000
H	6.77610000	-3.26730000	-2.00130000
H	5.85060000	-1.94550000	-2.79180000
C	6.56690000	1.87520000	2.11120000
H	7.41000000	1.28550000	2.49390000
H	6.82730000	2.93970000	2.11360000
H	5.67350000	1.70040000	2.71540000
C	-6.42260000	-1.97920000	2.06610000
H	-7.24180000	-1.41790000	2.53390000
H	-6.66030000	-3.04890000	2.06460000
H	-5.48440000	-1.79890000	2.59640000
C	-6.92920000	1.98000000	-2.23160000
H	-7.90550000	1.50300000	-2.38840000
H	-7.04940000	3.06880000	-2.20070000
H	-6.23420000	1.69650000	-3.02550000
H	-4.32350000	2.83420000	1.48930000
O	-6.01560000	4.86920000	-0.18850000

(R,R)-13

C	10.10310000	0.05790000	-3.38510000
C	9.43770000	-0.18710000	-2.20370000
C	8.50120000	0.74890000	-1.68100000
C	8.26690000	1.95460000	-2.42110000
C	8.96960000	2.17570000	-3.63570000
C	9.86970000	1.24990000	-4.11110000
H	10.81650000	-0.66980000	-3.76210000
C	7.78360000	0.52050000	-0.45990000
C	7.33160000	2.89800000	-1.92410000
H	8.78290000	3.09600000	-4.18400000
H	10.40240000	1.43070000	-5.04030000
C	6.67290000	2.68560000	-0.73350000
C	6.89930000	1.48510000	0.00110000
H	7.15880000	3.81280000	-2.48670000
C	7.94520000	-0.74760000	0.32160000
C	9.07620000	-0.94300000	1.17960000
C	6.96510000	-1.72620000	0.25060000
C	10.12110000	0.01430000	1.30660000
C	9.16440000	-2.14950000	1.94720000
C	7.01960000	-2.91130000	1.04930000
C	11.19560000	-0.21980000	2.13650000
C	10.28760000	-2.35940000	2.79350000

C	8.11900000	-3.09530000	1.86560000
C	11.28530000	-1.41760000	2.88630000
H	11.98390000	0.52380000	2.21750000
H	10.33840000	-3.28080000	3.36800000
H	8.15780000	-4.00160000	2.46150000
H	12.14010000	-1.58610000	3.53520000
O	5.88460000	-1.52460000	-0.60220000
O	6.13950000	1.26330000	1.12180000
C	5.75290000	3.73800000	-0.16300000
O	6.03380000	4.37400000	0.84380000
N	4.60490000	3.92360000	-0.90110000
H	4.46040000	3.26160000	-1.65240000
C	5.96990000	-4.00110000	1.13590000
O	6.21260000	-5.04540000	1.73390000
N	4.75930000	-3.72790000	0.54620000
H	4.70060000	-2.82490000	0.08340000
C	3.62680000	-4.55810000	0.47080000
C	3.45460000	-5.71910000	1.23760000
C	2.60810000	-4.18740000	-0.42160000
C	2.29260000	-6.47580000	1.10550000
H	4.23340000	-6.02840000	1.91930000
C	1.45520000	-4.95290000	-0.54450000
H	2.72390000	-3.29480000	-1.03240000
C	1.26380000	-6.10850000	0.22780000
H	2.20860000	-7.36960000	1.70910000
H	0.69410000	-4.64260000	-1.25170000
C	3.51940000	4.78050000	-0.62660000
C	3.58970000	5.84760000	0.27760000
C	2.31620000	4.55010000	-1.30950000
C	2.47430000	6.65860000	0.48420000
H	4.50720000	6.03390000	0.81690000
C	1.20800000	5.35390000	-1.08050000
H	2.24170000	3.72510000	-2.01490000
C	1.26070000	6.42350000	-0.17290000
H	2.57790000	7.48230000	1.17780000
H	0.28760000	5.13980000	-1.61150000
C	-1.26380000	-6.10850000	-0.22890000
C	-2.29260000	-6.47550000	-1.10660000
C	-1.45510000	-4.95310000	0.54390000
C	-3.45470000	-5.71880000	-1.23830000
H	-2.20870000	-7.36910000	-1.71050000
C	-2.60800000	-4.18760000	0.42130000
H	-0.69400000	-4.64310000	1.25110000
C	-3.62680000	-4.55800000	-0.47110000
H	-4.23360000	-6.02790000	-1.92000000
H	-2.72380000	-3.29530000	1.03250000
C	-1.26070000	6.42350000	0.17190000
C	-2.47420000	6.65830000	-0.48540000

C	-1.20800000	5.35420000	1.07980000
C	-3.58960000	5.84730000	-0.27850000
H	-2.57780000	7.48170000	-1.17930000
C	-2.31610000	4.55040000	1.30910000
H	-0.28750000	5.14040000	1.61090000
C	-3.51930000	4.78050000	0.62600000
H	-4.50710000	6.03330000	-0.81790000
H	-2.24150000	3.72570000	2.01480000
N	-4.75940000	-3.72790000	-0.54610000
C	-5.97020000	-4.00110000	-1.13530000
H	-4.70060000	-2.82490000	-0.08320000
O	-6.21300000	-5.04520000	-1.73360000
N	-4.60470000	3.92360000	0.90080000
C	-5.75300000	3.73810000	0.16320000
C	-6.67280000	2.68570000	0.73400000
C	-7.33130000	2.89820000	1.92470000
C	-6.89930000	1.48520000	-0.00040000
C	-8.26650000	1.95470000	2.42200000
H	-7.15850000	3.81310000	2.48720000
C	-7.78350000	0.52050000	0.46070000
C	-8.96880000	2.17590000	3.63670000
C	-8.50080000	0.74910000	1.68200000
C	-9.86880000	1.25000000	4.11240000
H	-8.78210000	3.09620000	4.18500000
C	-9.43720000	-0.18700000	2.20500000
C	-10.10230000	0.05800000	3.38660000
H	-10.40130000	1.43090000	5.04170000
H	-10.81560000	-0.66980000	3.76380000
C	-7.01990000	-2.91130000	-1.04850000
C	-8.11950000	-3.09540000	-1.86440000
C	-6.96510000	-1.72610000	-0.24990000
C	-9.16500000	-2.14970000	-1.94570000
H	-8.15850000	-4.00180000	-2.46020000
C	-7.94530000	-0.74750000	-0.32060000
C	-10.28850000	-2.35960000	-2.79160000
C	-9.07650000	-0.94310000	-1.17830000
C	-11.28630000	-1.41790000	-2.88420000
H	-10.33940000	-3.28110000	-3.36600000
C	-10.12150000	0.01420000	-1.30500000
C	-11.19630000	-0.21990000	-2.13460000
H	-12.14120000	-1.58640000	-3.53280000
H	-11.98460000	0.52360000	-2.21540000
O	-6.13970000	1.26330000	-1.12130000
O	-5.88430000	-1.52440000	0.60240000
H	-10.06580000	0.93680000	-0.73700000
H	-9.62870000	-1.10230000	1.65440000
H	10.06550000	0.93680000	0.73850000
H	9.62920000	-1.10230000	-1.65310000

C	6.09340000	-1.99980000	-1.94330000
H	6.93810000	-1.48590000	-2.41040000
H	6.26820000	-3.08260000	-1.95310000
H	5.17700000	-1.77120000	-2.49200000
C	6.71980000	1.67570000	2.37230000
H	7.64880000	1.13030000	2.57070000
H	6.90230000	2.75380000	2.36990000
H	5.98100000	1.42470000	3.13600000
C	-6.09260000	-1.99940000	1.94370000
H	-6.93720000	-1.48540000	2.41100000
H	-6.26730000	-3.08220000	1.95370000
H	-5.17600000	-1.77050000	2.49210000
C	-6.72030000	1.67590000	-2.37160000
H	-7.64950000	1.13060000	-2.56980000
H	-6.90260000	2.75400000	-2.36920000
H	-5.98170000	1.42470000	-3.13550000
H	-4.45990000	3.26150000	1.65200000
O	-6.03430000	4.37420000	-0.84350000
C	0.00000000	7.30070000	-0.00060000
C	0.00000000	-6.96900000	-0.00070000
C	-0.07840000	8.22060000	-1.25580000
C	0.07840000	8.22100000	1.25430000
C	-0.31470000	-7.88470000	1.22020000
C	0.31480000	-7.88420000	-1.22210000
F	-1.08860000	8.84830000	1.47640000
F	1.02700000	9.17760000	1.14420000
F	0.36460000	7.50460000	2.36200000
F	1.08850000	8.84790000	-1.47810000
F	-0.36460000	7.50400000	-2.36330000
F	-1.02710000	9.17730000	-1.14590000
F	0.34070000	-7.16220000	-2.36070000
F	1.51180000	-8.48230000	-1.09780000
F	-0.59460000	-8.87240000	-1.39160000
F	0.59480000	-8.87280000	1.38940000
F	-1.51170000	-8.48290000	1.09570000
F	-0.34070000	-7.16320000	2.35910000
